# Plasmonic Alloys Enhanced Metabolic Fingerprints for the Diagnosis of COPD and Exacerbations

**DOI:** 10.1021/acscentsci.3c01201

**Published:** 2024-01-27

**Authors:** Haiyang Su, Yuanlin Song, Shouzhi Yang, Ziyue Zhang, Yao Shen, Lan Yu, Shujing Chen, Lei Gao, Cuicui Chen, Dongni Hou, Xinping Wei, Xuedong Ma, Pengyu Huang, Dejun Sun, Jian Zhou, Kun Qian

**Affiliations:** † State Key Laboratory of Systems Medicine for Cancer, School of Biomedical Engineering, Institute of Medical Robotics and Med-X Research Institute, Shanghai Jiao Tong University, Shanghai 200030, P. R. China; ‡ Department of Pulmonary and Critical Care Medicine, Shanghai Respiratory Research Institute, Zhongshan Hospital, Fudan University, Shanghai 200032, P. R. China; § Shanghai Key Laboratory of Lung Inflammation and Injury, 180 Fenglin Road, Shanghai 200032, P. R. China; ∥ Center of Emergency and Critical Medicine, Jinshan Hospital of Fudan University, Shanghai 201508, P. R. China; ⊥ Department of Respiratory and Critical Care Medicine, Shanghai Pudong Hospital, Fudan University, Shanghai 201399, P. R. China; # Clinical Medical Research Center, Inner Mongolia People’s Hospital, Hohhot 010017, Inner Mongolia, P. R. China; ∇ Inner Mongolia Key Laboratory of Gene Regulation of The Metabolic Disease, Inner Mongolia People’s Hospital, Hohhot 010017, Inner Mongolia, P. R. China; • Inner Mongolia Academy of Medical Sciences, Inner Mongolia People’s Hospital, Hohhot 010017, Inner Mongolia, P. R. China; ° Shanghai Minhang District Gumei Community Health Center affiliated with Fudan University, Shanghai 201102, P. R. China; ¶ Department of Respiratory and Critical Care Medicine, Inner Mongolia People’s Hospital, Hohhot 010017, P. R. China; ■ Shanghai Key Laboratory of Gynecologic Oncology, Renji Hospital, School of Medicine, Shanghai Jiao Tong University, Shanghai 200127, P. R. China

## Abstract

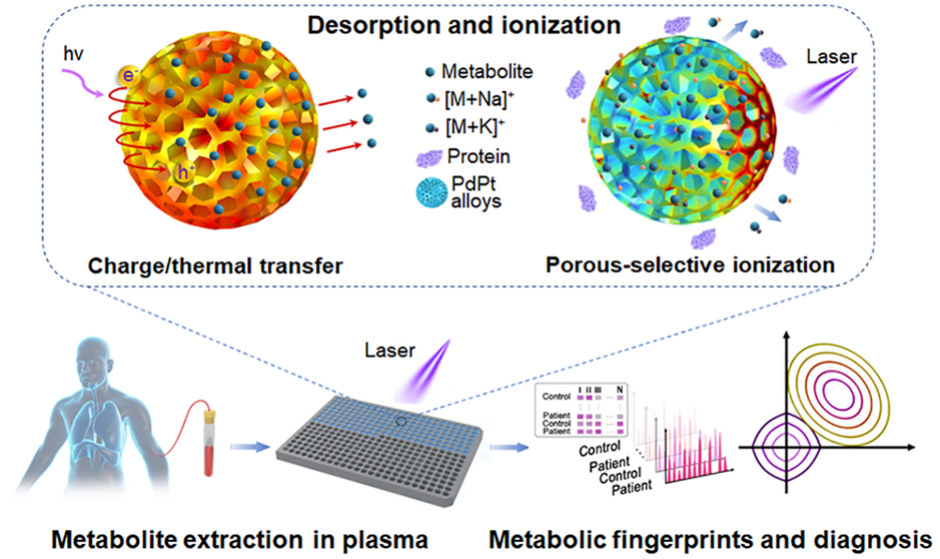

Accurate diagnosis of chronic obstructive pulmonary disease (COPD) and exacerbations by metabolic biomarkers enables individualized treatment. Advanced metabolic detection platforms rely on designed materials. Here, we design mesoporous PdPt alloys to characterize metabolic fingerprints for diagnosing COPD and exacerbations. As a result, the optimized PdPt alloys enable the acquisition of metabolic fingerprints within seconds, requiring only 0.5 μL of native plasma by laser desorption/ionization mass spectrometry owing to the enhanced electric field, photothermal conversion, and photocurrent response. Machine learning decodes metabolic profiles acquired from 431 individuals, achieving a precise diagnosis of COPD with an area under the curve (AUC) of 0.904 and an accurate distinction between stable COPD and acute exacerbations of COPD (AECOPD) with an AUC of 0.951. Notably, eight metabolic biomarkers identified accurately discriminate AECOPD from stable COPD while providing valuable information on disease progress. Our platform will offer an advanced nanoplatform for the management of COPD, complementing standard clinical techniques.

## Abstract 2

Plasmonic alloys enhanced metabolic fingerprints in plasma due to photoinduced charge/energy transfer and a size-exclusion effect, achieving a precise diagnosis of COPD and exacerbations.

## Introduction

Chronic obstructive pulmonary disease (COPD) is a prevalent condition characterized by persistent respiratory symptoms and airflow limitation and impacts approximately 300 million people globally.^1,2^ It is a major cause of morbidity and mortality worldwide, resulting in a global public health problem with mortality of about 3.2 million individuals annually.^2−4^ Advanced diagnostic methods could facilitate the development of subsequent treatment plans in time, improving the health outcomes of patients.^5,6^ However, COPD is a systemic condition, and spirometry, which is a commonly used clinical method for COPD diagnosis through the assessment of lung function alone, is still limited by its poor accuracy, accessibility, and patient compliance.^7,8^ Moreover, this method fails to provide information on the disease’s progress. In particular, there are no objective clinical tools available for the diagnosis of COPD exacerbations, which are the main factors of hospitalizations and mortality in COPD.^1,8,9^ Therefore, a reliable and noninvasive method, realized through robust analytical platforms for the diagnosis of COPD and exacerbations, is highly required.

Biomarkers allow the characterization of disease progression at the molecular level through noninvasive techniques,^10,11^ holding promise for the diagnosis of COPD and exacerbations.^9,12,13^ Recently, various gene (e.g., circulating miRNA)^14,15^ and protein biomarkers (e.g., C-reactive protein)^16,17^ have been reported for the diagnosis or evaluation of COPD, but their performance is suboptimal for clinical use due to their poor accuracy.^13^ Compared with genes and proteins, metabolites function as immediate indicators of biochemical activity and exhibit a close correlation with the COPD phenotype.^6,13^ Mass spectrometry (MS), specifically laser desorption/ionization (LDI) MS, has emerged as a robust analytical instrument for the high-throughput and sensitive detection of various metabolites.^18−24^ However, metabolic analysis is often impeded by the inherent challenges of concentration and purification, given the low concentration of metabolites and high complexity of samples in clinical specimens.^10,25−27^

Matrix materials play crucial roles in analyte detection, deciding the performance of LDI MS.^28−31^ In particular, noble metals are superior candidates for enhancing LDI efficiency, concerning the surface plasmon resonance and hot carriers generated under laser irradiation.^32,33^ However, most of the current matrixes focus on monometallic nanoparticles (e.g., Au, Ag, Pt, and Pd)^34,35^ or their composites with other materials,^36−38^ exhibiting insufficient sensitivity in LDI MS for clinical application. Additionally, there are very few studies investigating the relationship between particle size and selective LDI. Furthermore, nanoparticles with porous structures enable the enrichment of small metabolites and the exclusion of proteins, achieving selective ionization of metabolites.^22,39^ Therefore, bimetallic alloys with synergistic effects and mesoporous structure promise to alleviate the constraints of monometallic/metal-composite and nonporous matrixes and advance metabolite analysis toward precise diagnosis.

**Figure 1:**
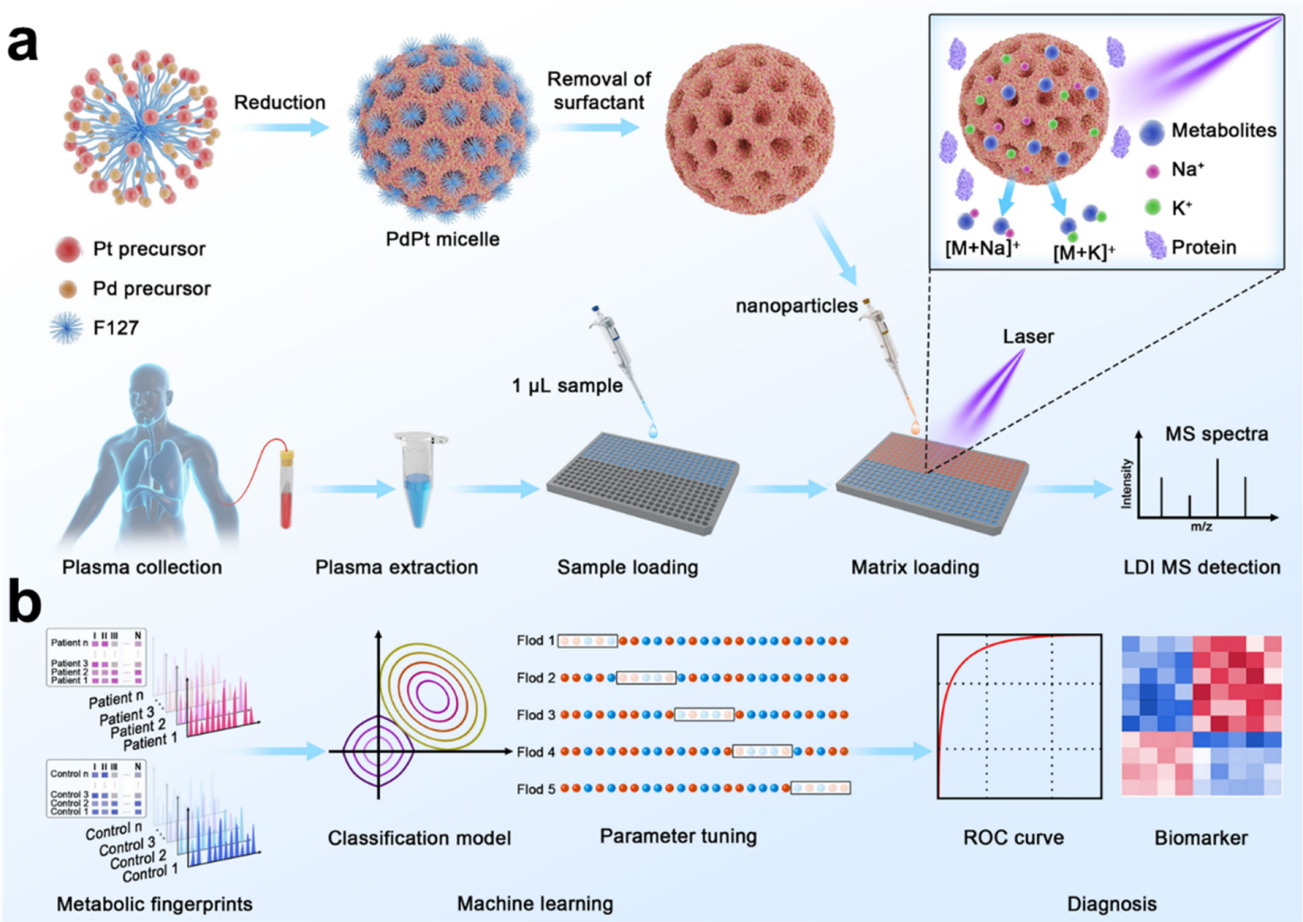
Schematics for extraction of plasma metabolic fingerprints toward the diagnosis of chronic obstructive pulmonary disease (COPD) and exacerbations. (a) Experimental procedure for the extraction of plasma metabolic fingerprints via laser desorption/ionization mass spectrometry (LDI MS) assisted by PdPt alloys. Mesoporous PdPt alloys were synthesized by utilizing F127 surfactant as a pore-directing template. One microliter of plasma extract (0.5 μL of native plasma) was used to obtain signals of metabolites with cation adducts (Na^+^/K^+^). (b) Schematic diagram for machine learning of metabolic fingerprints for diagnosis of COPD and exacerbations.

In this study, we constructed mesoporous PdPt-assisted LDI MS for metabolite profiling from plasma (Figure 1a), following the diagnosis of COPD and exacerbations and biomarker discovery via machine learning (Figure 1b). The excellent LDI performance of PdPt alloys was attributed to the enhanced electric field, photothermal conversion, photocurrent response, and mesoporous structure with size exclusion effect. The optimized PdPt alloys facilitated rapid metabolite profiling within seconds, requiring only 0.5 μL of native plasma without tedious pretreatment. By integration with machine learning, we achieved a precise diagnosis of COPD with an area under the curve (AUC) of 0.904 in the discovery cohort and an AUC of 0.955 in the validation cohort. Notably, our platform enables an accurate distinction between stable COPD (SCOPD) and acute exacerbations of COPD (AECOPD), with an AUC of 0.951 in the discovery cohort and an AUC of 0.976 in the validation cohort. Furthermore, eight identified metabolic biomarkers revealed a distinct signature for the discrimination of SCOPD and AECOPD. Our work provides an advanced nanoplatform for the precise diagnosis of COPD and exacerbations and valuable information on disease progress.

## Results and Discussion

### Synthesis and Characterization of Mesoporous PdPt Alloys

**Figure 2:**
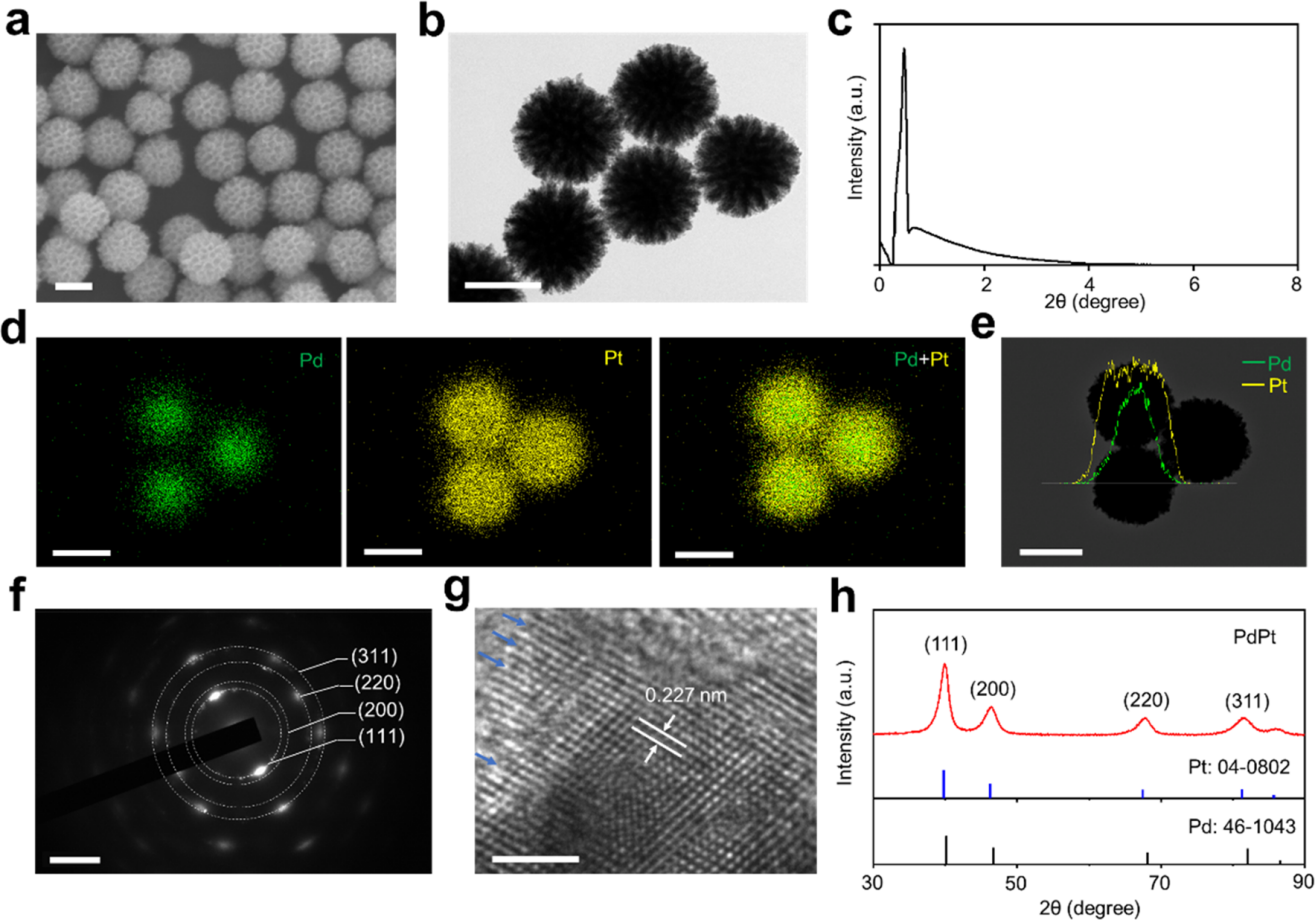
Characterization of PdPt alloys. Electron micrograph images of PdPt alloys, including (a) scanning electron microscopy (SEM) and (b) transmission electron microscopy (TEM). (c) Low-angle X-ray diffraction (XRD) pattern of PdPt with a sharp peak at 2θ = 0.46°. (d) Elemental mapping analysis and (e) line-scan energy-dispersive X-ray (EDX) results of PdPt alloys, with Pd in green and Pt in yellow, respectively. (f) Selected-area electron diffraction (SAED) pattern and (g) high-resolution TEM (HRTEM) of PdPt alloys. The arrows in (g) show the unsaturated Pt atoms. (h) Wide-angle XRD pattern of PdPt alloys, standard Pd (JCPDS: 46-1043) and Pt (JCPDS: 04-0802). Scale bar: (a, b, d, e) 100 nm for SEM, TEM, and mapping results, (f) 5/nm, and (g) 2 nm for HRTEM.

We successfully synthesized mesoporous PdPt spheres through a facile and modified surfactant-directing method.^22^ Briefly, the bimetallic alloys were obtained by the ascorbic acid-triggered reduction of Na_2_PdCl_4_ and H_2_PtCl_6_ in an aqueous solution, followed by the removal of F127 serving as pore-directing agents. Scanning electron microscopy (SEM) and transmission electron microscopy (TEM) images (Figure 2a,b) showed that the alloys were fairly uniform and possessed well-defined mesoporous structures with a pore size of approximately 20 nm. This was further characterized by low-angle X-ray diffraction (XRD) (Figure 2c) that showed a clear, sharp peak at 2θ = 0.46° (d = 19.0 nm), demonstrating the formation of a periodic mesoporous structure. The nitrogen adsorption–desorption isotherm of PdPt alloys exhibited a typical type-IV curve (Figure S1a) with a pronounced capillary condensation phenomenon at the relative pressure (P/P_0_) of 0.7–0.9, further evidencing the existence of a mesoporous structure.^40,41^ The specific surface area was ∼23.29 m^2^ g^–1^ as counted by the Brunauer–Emmett–Teller (BET) model. The pore size was ∼19.6 nm as calculated by the pore-size distribution curve (Figure S1b) based on the Barret–Joyner–Halenda (BJH) mode, which is almost consistent with the low-angle X-ray diffraction analysis. In particular, the mesopores on the alloy surface exhibited concavity toward the center of the particles, which was crucial to trapping small molecules while excluding larger nucleic acids and proteins in complex biofluids.^22,42^

Furthermore, elemental mapping and line scan profiles were investigated to reveal the distributions of Pd and Pt in the particles. As shown in Figure 2d, elemental mapping showed a pronounced concentration of Pd within the core region of the particles, while Pt was found to be uniformly distributed across the entirety of the spherical structure. This was further revealed by the line scan profiles (Figure 2e). The selected-area electron diffraction (SAED) pattern (Figure 2f) and high-resolution TEM (Figure 2g) with clear lattice fringes demonstrated the high degree of crystallization of the bimetallic alloys.^22,43^ The crystal structure was evidenced by the wide-angle XRD (Figure 2h), yielding (111), (200), (220), and (311) diffraction peaks of face-centered-cubic (fcc) crystal structure assignable to Pd (JCPDS: 46-1043) and Pt (JCPDS: 04-0802).^43,44^ X-ray photoelectron spectroscopy (XPS) (Figure S2) showed doublet peaks of Pt0 4f_7/2_ and Pt0 4f_5/2_ as well as peaks of Pd 3f_5/2_ and Pd 3f_3/2_, respectively, evidencing the presence of metallic state Pt and Pd on the surface of the alloys.^43,45^ The surface Pt/Pd mole ratio of porous PdPt was calculated to be 6.3, demonstrating that the surface of PdPt is abundant in Pt, consistent with the elemental mapping result (Figure 2d,e). Notably, the PdPt alloys demonstrated an fcc crystal structure with an elevated density of surface atoms with low coordination, possessing high surface energy.^46,47^ In particular, the unsaturated Pt atomic structures (with some atomic edge and kink sites as shown in Figure 2g) on the surface can provide sufficient active sites and enhance analyte adsorption and charge transfer to analytes,^43,48^ which will be beneficial for the following LDI process enhancement.

### Optimization of Mesoporous PdPt Alloys

**Figure 3:**
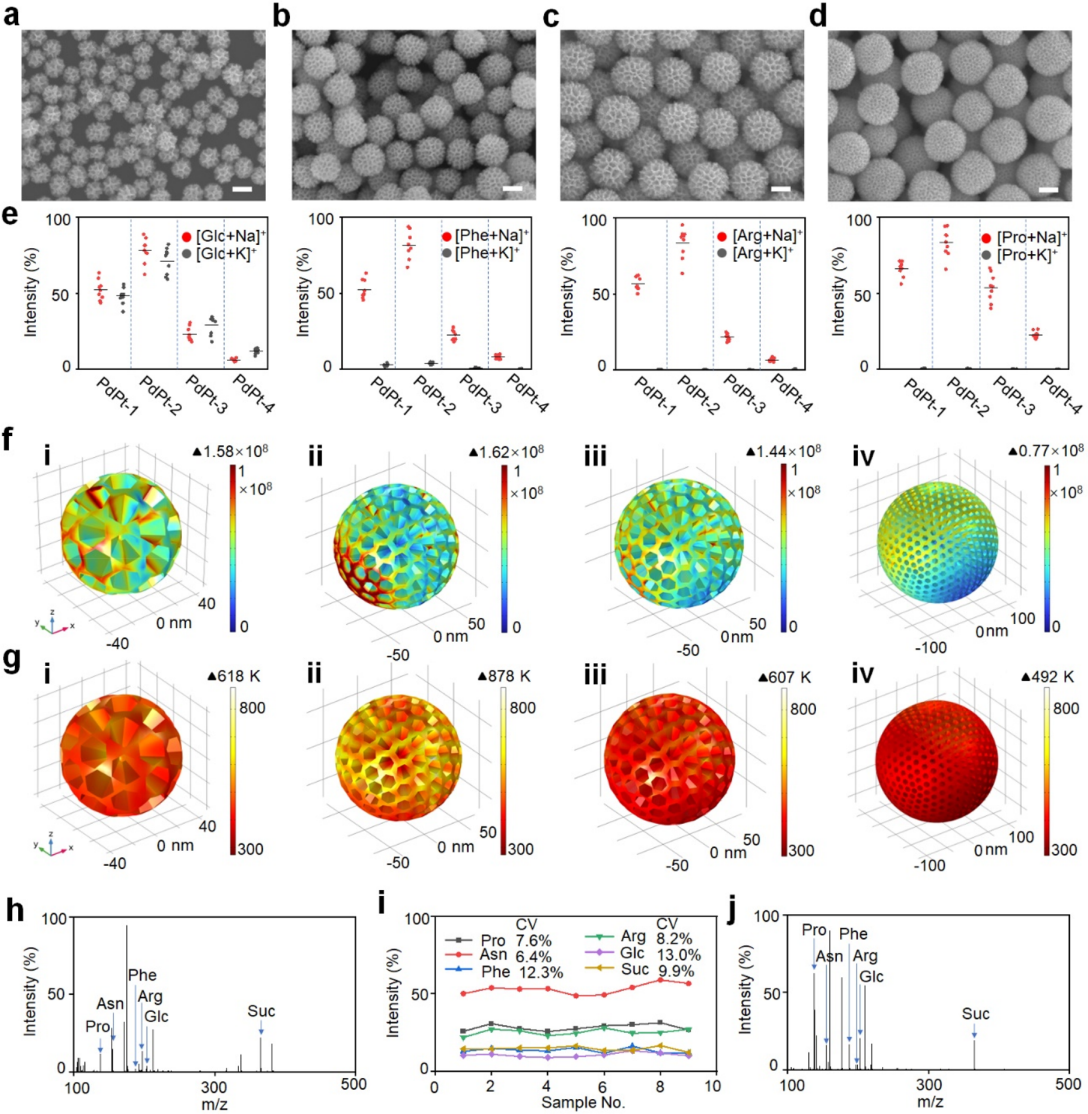
Optimization of PdPt alloys for metabolite detection. SEM images of (a) PdPt-1, (b) PdPt-2, (c) PdPt-3, and (d) PdPt-4 alloys with different particle sizes. (e) Intensities of sodium- and potassium-adducted signals of glucose (Glc), phenylalanine (Phe), arginine (Arg), and proline (Pro) detected by PdPt-1/2/3/4 alloy-assisted LDI MS. The error bars were determined as the standard deviation (s.d.) of nine measurements. Contour plots of (f) electric field amplitudes and (g) thermal field distribution shown on the color scale for (i) PdPt-1, (ii) PdPt-2, (iii) PdPt-3, and (iv) PdPt-4 alloys, for a 355 nm laser beam with polarization along the *Y* axis. Laser light was introduced along the *Z*-axis. The electric field amplitudes and thermal field distribution were counted by the finite element method. (h) Typical mass spectrometry of different metabolites (Pro, asparagine (Asn), Phe, Arg, Glc and sucrose (Suc)) in water solution, detected by PdPt-2 alloys assisted LDI MS. (i) Coefficient of variance (CV) distribution of different metabolites (Pro, Asn, Phe, Arg, Glc and Suc) analyzed by PdPt-2 alloys assisted LDI MS. The results come from nine independent experiments. (j) Typical mass spectrometry of different metabolites (Pro, Asn, Phe, Arg, Glc, and Suc) in a mixture solution of 0.5 M NaCl (salt tolerance detection) and 5 mg mL^–1^ bovine serum albumin (protein endurance detection), detected by PdPt-2 alloy-assisted LDI MS. Scale bar: (a, b, c, d) 100 nm.

LDI enhancement of nanomaterials was proven to be closely correlated with their elemental compositions and surface structures by numerous studies.^22,49^ However, rarely have reports demonstrated the effects of nanomaterial size on the LDI process. Therefore, we synthesized mesoporous PdPt nanoparticles with controlled particle size by changing the concentration of HCl (3 M (250 μL)/6 M (250 μL)/12 M (250 μL)/12 M (500 μL) in HCl aqueous solution, denoted as PdPt-1/2/3/4). As the HCl concentration increased, we observed increased particle size with an average size of approximately 90/150/200/240 nm for PdPt-1/2/3/4 alloys (Figure 3a–d and Figure S3). Energy-dispersive X-ray (EDX) analysis revealed similar Pd and Pt contents for PdPt-1/2/3/4 alloys (Figure S4 and Table S1), which was helpful in investigating the effect of particle size on the LDI process without the influence of elemental composition. The zeta potential demonstrated that the surface of PdPt-1/2/3/4 was negatively charged and the charge increased from −44.07 ± 0.65 mV to −22.77 ± 0.60 mV as the particle size increased (Figure S5 and Table S2), which is helpful for cation adduction and matrix use in LDI MS.^22,49,50^ All PdPt nanospheres exhibited strong absorption in the ultraviolet (UV) range (Figure S6). Considering the wavelength (355 nm) of the Nd:YAG laser used for LDI MS, mesoporous PdPt alloys may allow efficient laser energy transfer to analytes.^22,51^

To select the optimized materials for the following biofluids detection, a thorough LDI MS performance evaluation of the PdPt nanoparticles was conducted for glucose (Glc), phenylalanine (Phe), arginine (Arg), and proline (Pro) analysis. As a result, PdPt-2 presented significantly elevated signal intensities (*p* < 0.05) for different small molecules, superior to PdPt-1/3/4 alloys (Figure 3e). In particular, PdPt-2 alloys still exhibited higher signal intensity (*p* < 0.05) of glucose compared with that of other materials for plasma detection (Figure S7), indicating PdPt-2 alloys to be the matrix for the ideal detection of metabolites in clinical samples. Furthermore, various kinds of metabolites (30 small molecules) including nonpolar amino acids, polar amino acids, carbohydrates, alkaloids, nucleotides, fatty acids, and organic acids were used to comprehensively evaluate the LDI performance of PdPt-2 alloys. Consequently, PdPt alloys showed preferred performance for polar amino acids and carbohydrates (Figure S8) because metabolites with hydrophilic chemical groups (such as hydroxyl groups) can be desorbed and ionized on the surface of PdPt alloys with homogeneous cocrystallization.^52,53^ The superior LDI performance of PdPt-2 alloys was assigned to the enhancement of the electric field for desorption/ionization induced by light, photothermal conversion for desorption triggered by heat, and a photocurrent response for the inhibition of electron–hole recombination.

To demonstrate the electric field enhancement, a three-dimensional finite element simulation was conducted to model the electric/thermal field distribution when exposed to a 355 nm wavelength laser matched with the laser source of LDI MS. As a result, PdPt-2 offered the greatest electric field (shown as E^2^) of 1.62 × 10^8^ (Figure 3f), a maximum ∼2.1-fold higher over PdPt-1/3/4 due to its unique particle size. Notably, porous bimetallic alloys displayed a superior electric field enhancement of 1.3–2.4-fold compared to that of porous/nonporous monometallic particles (Figure S9a), attributed to the facile charge transfer among multiple elements and porous structure.^22,54^ Specifically, a PdPt alloy with noble metal characteristics may generate hot carriers and strong optical near-field effects under UV (355 nm) illumination.^32,55^ Even though the resonance wavelengths of both Pd and Pt solid nanoparticles are not 355 nm, the PdPt alloy can generate a strong plasmonic effect under UV illumination due to the special alloy properties and porous structure. The alloy structure may lead to interactions between Pd and Pt, creating surface-isolated exciton coupling effects and altering the electromagnetic response of the single material.^56,57^ Furthermore, the nanopores act as resonators, concentrating the incoming electromagnetic field and generating high-density hotspots, despite the fact that the plasmons are not in resonance under short-wavelength irradiation.^54,58^ Thus, the surface adsorbates on the alloys are expected to generate an enhanced MS signal due to effective desorption/ionization induced by hot carriers that are triggered by near-field enhancement.^22,59^

For the photothermal conversion, a three-dimensional finite element simulation was first carried out to study the temperature of PdPt alloys under a 355 nm wavelength. The highest temperature reached 878 K for PdPt-2, higher than for PdPt-1/3/4 bimetallic alloys and monometallic particles (Figure 3g and Figure S9b), owing to its distinct particle size and lower thermal conductivity compared to that of monometals.^60^ The bimetallic alloys with lower thermal conductivity could minimize the diffusion of thermal energy and thus were heated to a higher temperature under the same laser irradiation fluence,^60,61^ leading to higher photothermal efficiency and the efficient desorption of nearby analytes compared to monometallic particles. The rapid relaxation processes of hot carriers produced by metals can cause local heating, resulting from the plasmon resonances, to boost light absorption in the surface area of the metal, consequently enhancing the conversion of absorbed light energy.^62,63^ In addition, the light absorption of a nonplanar (like porous) metal surface is more efficient than that of planar structures that reflect most of the incident light.^62^ Notably, the high temperature facilitating the partial melting of materials induced surface structural changes at high fluence areas (Figure S10), improving the thermal desorption and phase transition processes while facilitating the desorption/ionization of analytes.^22,55,64^ Although there are no significant differences in the pore diameter between PdPt-2 and PdPt-1/3 alloys, significant differences exist between PdPt-2 and PdPt-4 alloys (Figure S11a). We further compared the electric field and photothermal conversion of PdPt-2 and PdPt-4 alloys without significant differences in pore size. The PdPt-4 alloy model with a 20 nm pore size was built for COMSOL simulation, still exhibiting a lower maximum electric field (shown as E2) of 0.85 × 10^8^ and a temperature of 513 K compared to those of PdPt-2 alloys (Figure S11b,c). For the photocurrent response, the PdPt-2 alloy displayed the strongest photocurrent intensity compared to PdPt-1/3/4 alloys (Figure S12), indicating a reduced rate of electron–hole recombination.^51^ Considering the distinctive optical, electric, and thermal characteristics, the PdPt-2 alloys offer notable advantages in the highly sensitive detection of metabolites.

To further demonstrate the advantage of the PdPt-2 matrix over traditional organic matrixes (α-cyano-4-hydroxycinnamic acid (CHCA) and 2,5-dihydroxybenzoic acid (DHB)), we compared the detection reproducibility in a mixture of six metabolites, including Pro, asparagine (Asn), Phe, Arg, Glc, and sucrose (Suc). Typical mass spectrometry of different metabolites is shown in Figure 3h. The intensity coefficients of variation (CVs) for six molecules were 7.6%, 6.4%, 12.3%, 8.2%, 13.0%, and 9.9%, respectively (Figure 3i), superior to CHCA and DHB with CV values of over 25.0% (Figure S13a,b). The excellent reproducibility of the PdPt-2 alloy benefited from the more homogeneous nanoparticle–analyte cocrystals, compared to the random sample crystallization with organic matrixes (Figure S13c–e).^18,33^ The limits of detection (LOD) for metabolites by PdPt alloys and organic matrixes were further studied by changing the molecule concentration from 100 to 0.1 μg mL^–1^. The regression equation exhibited excellent linear correlation results with R^2^ > 0.980 for each metabolite by PdPt alloy-assisted LDI MS analysis (Figure S14). The nanoplatform demonstrated high sensitivity detection for metabolites, with a LOD of as low as 0.3 pmol (Table S3). In contrast, organic CHCA and DHB matrixes showed poor linear correlation results with R^2^ = 0.697–0.975 (Figure S15). In particular, CHCA cannot detect the signals of Glc and Suc, and DHB cannot obtain the signals of Pro, Phe, Glc, and Suc, even at a high concentration of 100 μg mL^–1^ (Figure S15 and Table S3). Therefore, PdPt alloys outperform the organic matrix and exhibit lower limits of detection for small metabolite detection.

In light of metabolic abundance and sample complexity influencing the MS analysis, tedious pretreatment is essential for concentrating and segregating metabolites from complex biofluids.^65^ Nanoparticle with porous/crevices structure can concentrate metabolites in the nanopores for in situ macromolecule exclusion in biofluids, attaining a targeted LDI process of small metabolites.^22,66^ Consequently, the PdPt alloy with a porous structure realized the targeted and sensitive detection of metabolites (Figure 3j) in the presence of a high salt concentration (0.5 M NaCl) and protein content (5 mg mL^–1^ bovine serum albumin (BSA)). To further prove the size-selective effect in porous PdPt, glucose and BSA were used as model molecules for metabolites and protein, respectively, to observe the carbon element distribution in the alloy-analytes hybrids. The element mapping analysis revealed that the metabolites were captured by pores in alloy-metabolite hybrids, whereas such enrichment was not observed in the alloy-protein composites (Figure S16). Notably, we can observe a large number of small-molecule signal peaks in the detection of plasma, serum, and urine by PdPt-2 alloy-assisted LDI MS, outperforming CHCA and DHB as the matrix (Figure S17). Therefore, PdPt-2 alloys with optimized structure enabled the sensitive and selective detection of metabolites in complex biofluids toward clinical sample analysis.

### Enhanced Metabolic Fingerprints for COPD Diagnosis

**Figure 4:**
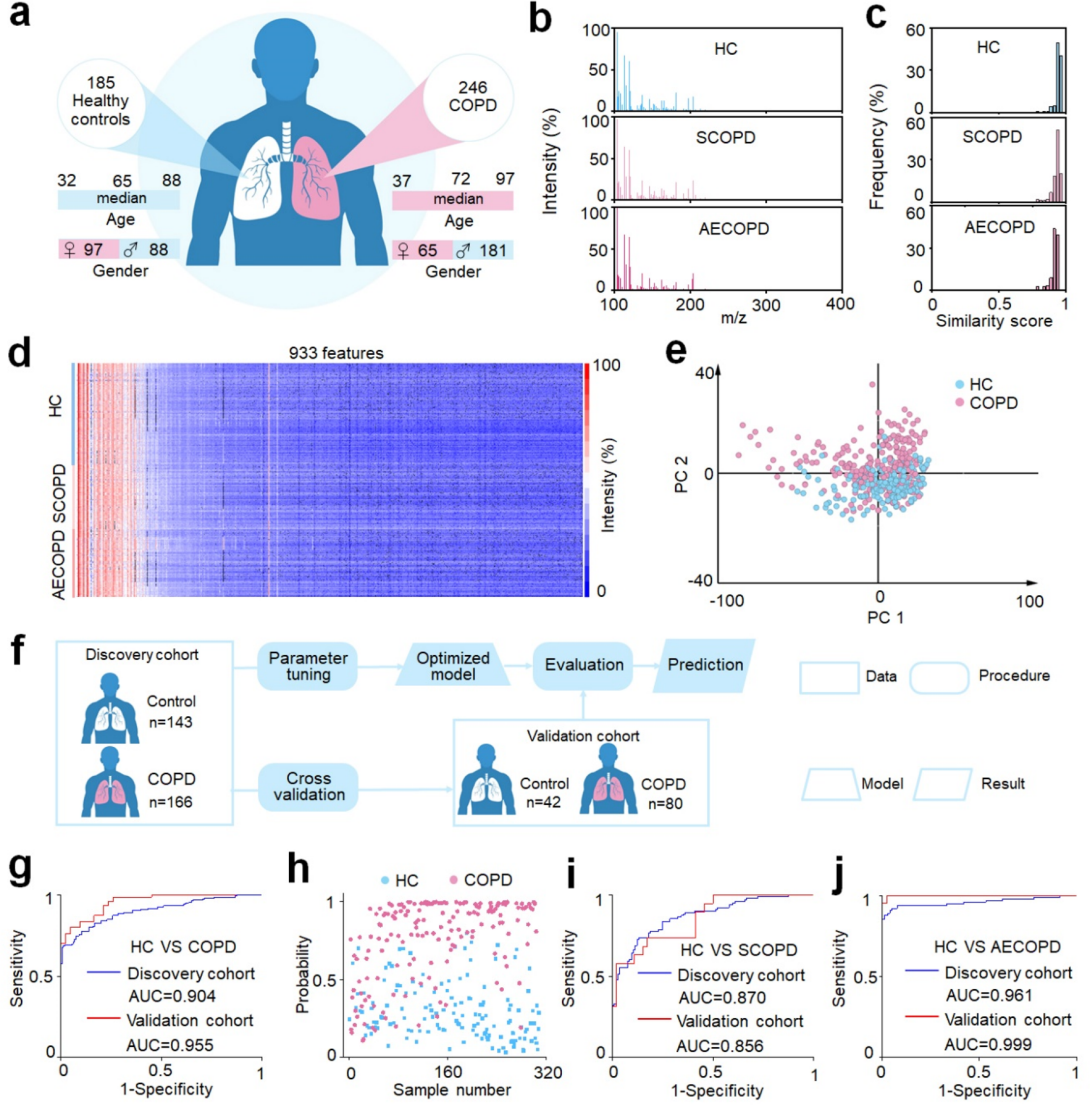
Machine learning of metabolic fingerprints for COPD diagnosis. (a) Demographic characteristics of 431 clinical specimens, including age and gender information on 185 healthy controls (HC) and 246 COPD patients (122 stable COPD (SCOPD) patients and 124 acute exacerbations of COPD (AECOPD) patients). (b) Typical mass spectra of plasma extracts from HC, SCOPD, and AECOPD samples with *m*/*z* ranging from 100 to 400, using 0.5 μL of native plasma. (c) The frequency distribution of similarity scores was computed for HC, SCOPD, and AECOPD groups. (d) Metabolic fingerprints were extracted from raw mass spectra of 185 healthy controls and 246 COPD patients, each containing 933 *m*/*z* features. (e) The unsupervised principal component analysis (PCA) showed a certain degree of discrimination between 185 healthy controls and 246 COPD patients. (f) Workflow for the diagnosis of COPD by machine learning. The discovery cohort comprised 309 samples (143/166, HC/COPD) used for parameter tuning and model construction. The optimized model was evaluated using an independent validation cohort with 122 subjects (42/80, HC/COPD). No statistically significant differences in age and gender between HC and COPD in the discovery cohort (*p* > 0.05). (g) The receiver operator characteristic (ROC) curve differentiates HC from COPD for the discovery (blue) and validation (red) cohorts. (h) Scatter diagram for HC and COPD from the discovery cohort. A probability of close to 1 implied a high level of certainty in the model that the sample belonged to class 1 (patient). In contrast, a probability close to 0 indicated a model inclination toward classifying the sample as class 0 (healthy control).^22,92^ ROC curves differentiate (i) HC from SCOPD and (j) HC from AECOPD for the discovery (blue) and validation (red) cohorts.

To uncover the specific metabolic signatures of COPD, we collected 431 plasma samples (Figure 4a) including 185 healthy controls and 246 COPD patients (122 SCOPD and 124 AECOPD individuals) for PdPt-2 alloy-assisted LDI MS analysis. The healthy controls were verified by spirometry with normal lung function (FEV1/FVC > 70%), and all patients were diagnosed with persistent airflow limitation based on the clinic criteria for the diagnosis of COPD.^67,68^ In this research, the test sample and the PdPt alloy suspension were prepared layer-by-layer (see details of Experimental Procedures in the Supporting Information) due to the high ionization efficiency.^22,69^ In contrast, the LDI performance showed a significant decrease (*p* < 0.05, Figure S18) if the alloy suspension was positioned ahead of the plasma sample or if the alloys and plasma were premixed. The metabolic fingerprints of plasma (Figure 4b) were successfully recorded in seconds in the low mass range (100–400 Da), with simple sample pretreatment and minimal sample consumption (0.5 μL of native plasma). Traditionally, liquid chromatography (LC) MS and nuclear magnetic resonance (NMR) are common methods for metabolite analysis, requiring tedious sample pretreatment (∼hours) and large sample consumption (∼milliliters).^65,70^ In contrast, PdPt-2 alloy-assisted LDI MS exhibited simple sample pretreatment (only protein precipitation required), improved detection speed, and minimized sample usage due to the enhanced sensitivity through optical/electrical/photothermal characteristics and in situ enrichment by the porous structure. In particular, over 95% of samples exhibited high similar scores over 0.85 for mass spectra in both the control and patient groups (Figure 4c), demonstrating the reliability of plasma metabolic fingerprints for further diagnostic applications. As a result, we obtained 933 *m*/*z* signals from the plasma via data processing (refer to the Experimental Procedures) and built the heat map composed of the metabolic data matrix of controls and patients (Figure 4d). However, we cannot see a significant difference between groups from the heat map. Furthermore, unsupervised analysis of the principle component analysis (PCA) showed a certain degree of separation without enough clarity between healthy controls and COPD patients (Figure 4e), indicating the imperative for developing a cutting-edge machine-learning method to find the distinct metabolic phenotype of COPD.

To further reveal the unique metabolic signature of COPD, sparse learning was performed for metabolic data analysis. Initially, we grouped the 431 plasma samples into a discovery cohort (143 healthy controls and 166 COPD patients) for parameter tuning and model optimization and an independent validation cohort (42 healthy controls and 80 COPD patients) for model evaluation and prediction (Figure 4f). No statistically significant differences in age and gender were observed between the two groups in the discovery cohort (*p* > 0.05, Table S4). Subsequently, we carried out the power analysis on a preliminary study of 12 samples (6/6, healthy control/COPD patient) to count the minimum required sample size for detecting a statistically significant difference (Figure S19). As a result, a predicted power of 0.92 was obtained at a false discovery rate (FDR) of 0.1 with a sample number of 48 (24/24, healthy control/COPD patient), evidencing the statistics of machine learning at a confidence level. A receiver operating characteristic (ROC) curve was graphed to illustrate the performance of sparse learning for discriminating COPD from healthy controls, producing an averaged area under the curve (AUC) of 0.904 with a 95% confidence interval (CI) of 0.869–0.939 (with a sensitivity of 0.801, specificity of 0.853, and accuracy of 0.825) for the discovery cohort (Figure 4g). Furthermore, we confirmed that there was no overfitting for the sparse learning model via a permutation test (Figure S20a, 1000 times and *p* < 0.001). Similarly, a consistent performance generated by the optimized model for the independent validation cohort was obtained, showing an AUC of 0.955 with 95% CI of 0.923–0.988 (with a sensitivity of 0.838, specificity of 0.833, and accuracy of 0.836). Notably, the possibility of each subject (in the discovery cohort) being diagnosed as a patient by the classification model was plotted as the scatter diagram that exhibited a clear separation of these two groups, indicating metabolic alteration accompanied by disease occurrence (Figure 4h). These results evidenced the advanced diagnostic power toward COPD by metabolic fingerprints through the PdPt alloy-assisted LDI MS analysis.

To search for significant metabolic biomarkers for COPD diagnosis, we screened top-ranking 4 *m*/*z* features based on the frequency, *p* value, abundance, and AUC of a feature produced by the sparse learning for discovery cohort analysis (Figure S21a). The features were glucose, lactic acid, uric acid, and malondialdehyde (Table S5), confirmed by the human metabolome database (HMDB) (http://www.hmdb.ca/), accurate MS measurement on Fourier transform ion-cyclotron resonance mass spectrometry (FT-ICR-MS), and identification by ultraperformance liquid chromatography-MS (UHPLC-MS) analysis (<5 ppm). In particular, glucose was down-regulated, and lactic acid, uric acid, and malondialdehyde were up-regulated in COPD patients (Figure S21b–e), constructing a classification model with an AUC of 0.779 (CI: 0.727–0.831) for COPD diagnosis in the discovery cohort (Figure S21f). Globally, the prevalence of COPD in males was about double that in females mainly due to common tobacco smoking among men,^1,71^ a similar phenomenon found in this work (Table S4). However, in the discovery cohort, the *p* value of gender was 0.43, indicating no significant difference in gender between healthy controls and the COPD patients. Therefore, differences in the number of males and females did not affect diagnostic modeling and biomarker discovery for COPD. Additionally, we compared the metabolic differences between males and females. However, there were no significant metabolic differences (*p* > 0.05) between males and females (Figure S22) for the four top-ranking significant metabolic biomarkers (including glucose, lactic acid, uric acid, and malondialdehyde) in COPD diagnosis, further demonstrating that the diagnostic model was not affected by gender.

To comprehensively evaluate the performance of our platform in COPD diagnosis, for both stable and acute exacerbations, sparse learning was conducted for the discrimination of healthy controls and SCOPD as well as healthy controls and AECOPD, respectively. No significant differences existed in age and gender for both discovery cohorts (*p* > 0.05, Tables S6 and Table S7). Consequently, we obtained an AUC of 0.870 (with 95% CI of 0.823–0.971) in the discovery cohort (105 healthy controls and 103 SCOPD patients) and an AUC of 0.856 (with 95% CI of 0.760–0.952) in the validation cohort (46 healthy controls and 19 SCOPD patients; Table S6) for the discrimination of healthy controls and SCOPD (Figure 4i). Meanwhile, we got an AUC of 0.961 (with 95% CI of 0.932–0.991) in the discovery cohort (105 healthy controls and 102 AECOPD patients) and an AUC of 0.999 (with 95% CI of 0.995–1) in the validation cohort (46 healthy controls and 22 AECOPD patients; Table S7) for the distinction between healthy controls and AECOPD (Figure 4j). These results further demonstrated that the constructed PdPt alloy-assisted LDI MS platform was capable of achieving a precise diagnosis of COPD.

Clinically, the diagnosis of COPD is confirmed by the existence of consistent airflow limitation, as evaluated by postbronchodilator spirometry.^1,67^ However, this method generally underestimates the prevalence of COPD in younger patients and overestimates it in older patients.^72^ In particular, this method presents poor patient compliance for people with underlying diseases and acute exacerbations. Currently, there is a deficiency in reliable genes or protein biomarkers to assess the diagnosis of COPD.^15,17,73^ In contrast, metabolite biomarkers function as direct indicators of biochemistry activity, closely correlating the phenotype of disease.^74^ Here, we developed PdPt alloy-assisted LDI MS for the effective extraction of metabolic fingerprints in plasma due to the unique physiochemical property and porous structure. Notably, the AUC for the discrimination of healthy controls and COPD patients is beyond 0.90, demonstrating the dependability and application potential of metabolic fingerprints for COPD diagnosis.

### Biomarker Discovery for AECOPD Diagnosis

**Figure 5:**
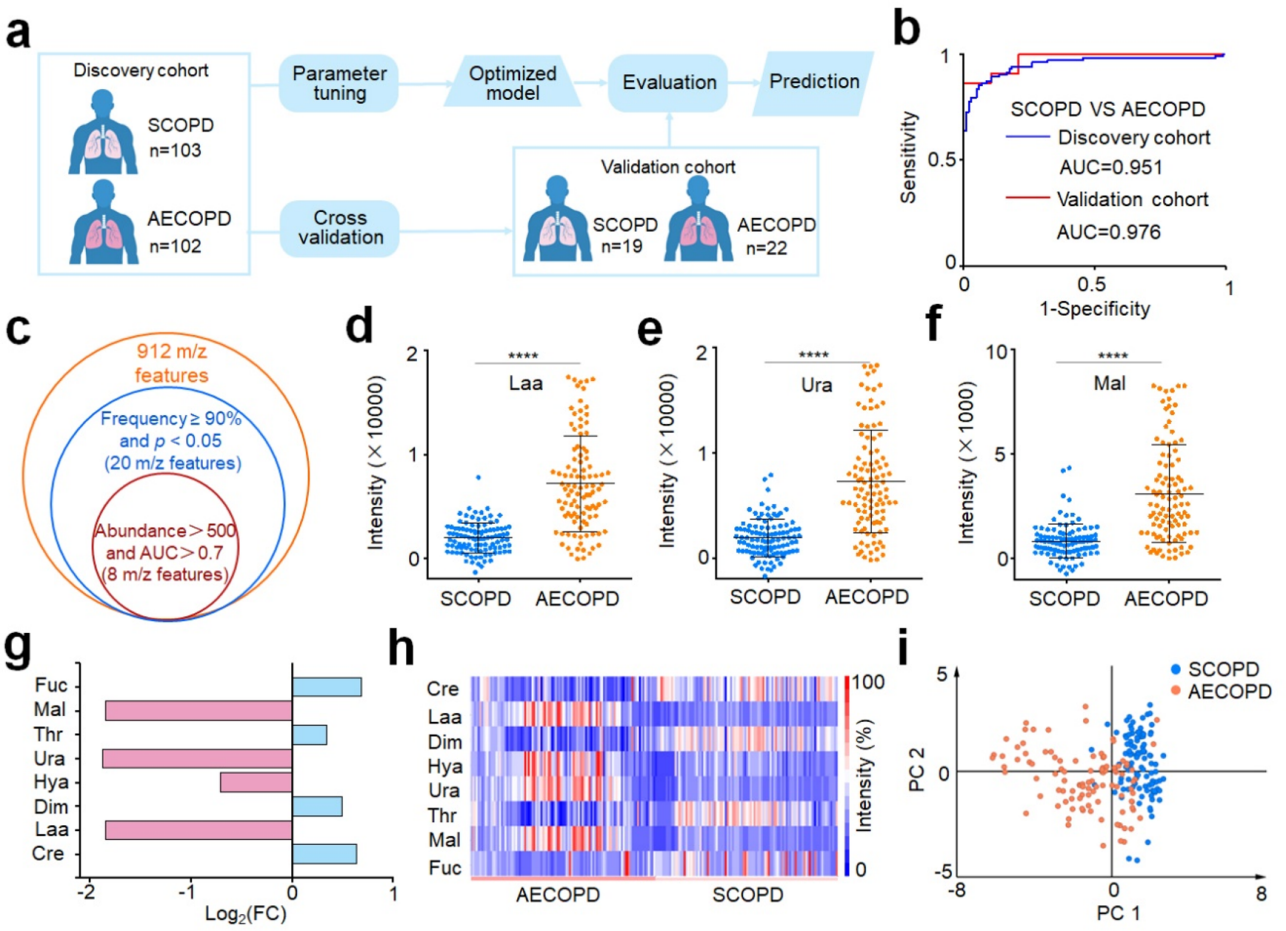
Machine learning of metabolic fingerprints for AECOPD diagnosis and biomarker discovery. (a) Workflow for the diagnosis of AECOPD by machine learning. The discovery cohort included 205 samples (103/102, SCOPD/AECOPD) for parameter tuning and model construction. The optimized model was tested in an independent validation cohort with 41 individuals (19/22, COPD/AECOPD). No statistically significant differences in age and gender between SCOPD and AECOPD in the discovery cohort (*p* > 0.05). (b) ROC curves differentiate SCOPD from AECOPD for the discovery (blue) and validation (red) cohorts. (c) Venn diagram of 8 *m*/*z* features screened as the metabolic signature panel with frequency ≥90%, *p* < 0.05, abundance >500, and AUC of single feature >0.7. Scatter diagram of three key differential features for SCOPD and AECOPD, including (d) lactic acid (Laa), (e) uric acid (Ura), and (f) malondialdehyde (Mal). **** is represented by *p* < 0.0001. (g) Fold change of four up-regulated metabolites (Laa, Ura, Mal, and 3-hydroxybutyric acid (Hya) with magenta color) and four down-regulated metabolites (creatine (Cre), dimethylglycine (Dim), threonine (Thr), and fucose (Fuc) with cyan color) in AECOPD patients compared with SCOPD. (h) The heat map of the discovery cohort, including SCOPD and AECOPD patients, is constructed by eight metabolic biomarkers as potential signatures for AECOPD diagnosis. (i) PCA analysis showed a clear discrimination between SCOPD and AECOD patients based on eight metabolic biomarkers.

AECOPD features acute worsening of respiratory symptoms and is the primary cause of mortality from COPD,^68,75^ indicating that early diagnosis is important for patient survival and prognosis. To construct powerful tools for the diagnosis and assessment of AECOPD, we randomly grouped the 246 (122 SCOPD and 124 AECOPD patients) plasma samples into a discovery cohort (103 SCOPD and 102 AECOPD patients) for parameter tuning and model optimization and an independent validation cohort (19 SCOPD and 22 AECOPD patients) for model evaluation and prediction (Figure 5a). No statistically significant differences in age and gender were observed between the two groups in the discovery cohort (*p* > 0.05, Table S8). After obtaining the metabolic fingerprints of all plasma samples by PdPt alloy-assisted LDI MS, we extracted 912 *m*/*z* metabolic signals in plasma via data processing. Unsupervised PCA based on all of these signals was plotted to demonstrate the difference between SCOPD and AECOPD, yielding an unclear separation between the two groups (Figure S23a). We further performed sparse learning for the metabolic data analysis, achieving a precise diagnosis of AECOPD with an AUC of 0.951 (with 95% CI of 0.920–0.982, sensitivity of 0.852, specificity of 0.942, and accuracy of 0.898) in the discovery cohort (Figure 5b). Furthermore, we confirmed that there was no overfitting for the sparse learning model via a permutation test (Figure S20b, 1000 times and *p* < 0.001). Additionally, based on the optimized model, a comparable AUC of 0.976 (with 95% CI of 0.939-1, a sensitivity of 1, a specificity of 0.789, and an accuracy of 0.902) was obtained in the independent validation cohort (Figure 5b). The above results prove that our platform can realize the precise diagnosis of AECOPD, showing great potential in the management of AECOPD. SCOPD and AECOPD represent different stages of COPD’s disease course with different clinical features and treatment needs.^68,76^ In particular, AECOPD is a progressive stage of stable COPD (SCOPD), with a high rate of hospitalization and mortality. Distinguishing between them helps to tailor individualized treatment regimens more precisely, improving survival and prognosis.^77^ Furthermore, the onset of AECOPD accompanies the changes in biomarkers in the blood, and discriminating SCOPD and AECOPD will help to develop significant metabolic biomarkers for the early diagnosis and assessment of AECOPD progression.^1,78^

In clinical situations, there are no reliable biomarkers with enough sensitivity and specificity to predict AECOPD onset. To search for the distinct metabolic biomarkers between SCOPD and AECOPD, we screened top-ranking 8 *m*/*z* features based on the frequency, *p* value, abundance, and AUC of a single feature produced by the sparse learning for discovery cohort analysis (Figure 5c). These features were confirmed for eight metabolites according to the HMDB (http://www.hmdb.ca/), accurate MS measurement on FT-ICR-MS, and identification by UHPLC-MS analysis (<5 ppm). These biomarkers included creatine (Cre), lactic acid (Laa), dimethylglycine (Dim), 3-hydroxybutyric acid (Hya), uric acid (Ura), threonine (Thr), malondialdehyde (Mal), and fucose (Fuc). The scatter diagram demonstrated that four metabolites (Laa, Hya, Ura, and Mal) were up-regulated and four metabolites (Cre, Dim, Thr, and Fuc) were down-regulated in AECOPD compared with COPD (Figure 5d–f and Figure S24). This was further validated by the fold-change map, in which Laa, Hya, Ura, and Mal showed relatively high fold changes (Figure 5g). The heat map established with the eight-biomarker panel presented a noticeable distinction between SCOPD and AECOPD, offering proof of the promising metabolic assessment of AECOPD (Figure 5h). PCA of these eight metabolites (Figure 5i) displayed enhanced separation of SCOPD and AECOPD, compared with that of all 912 *m*/*z* features (Figure S23a). Notably, the diagnostic performance of the combination of eight biomarkers outperformed any single biomarker (Table S9), affording an enhanced AUC of 0.904 (95% CI of 0.859–0.950) for the discovery cohort and an AUC of 0.955 (95% CI of 0.874–1) for the validation cohort (Figure S23b). These results demonstrate that the eight screening metabolic biomarkers can accurately distinguish SCOPD from AECOPD, providing promising biomarkers for the diagnosis of COPD exacerbations and the assessment of COPD progress.

AECOPD often requires emergency department care and is also the primary cause of death from COPD.^1,67^ Ideal biomarkers facilitate the evaluation of the risk of exacerbation for individuals with stable COPD. Generally, C-reactive protein (CRP) is used for the diagnosis of AECOPD or the prediction of the frequency and severity of exacerbations.^9,79^ However, CRP is neither sufficiently sensitive nor specific, showing an AUC of 0.73 for the AECOPD diagnosis.^80^ In addition, fibrinogen and the leukocyte count are frequently used for the evaluation of risk in exacerbations, exhibiting poorer diagnostic results than the CRP biomarker.^79,80^ In contrast, the AUCs of the eight metabolic biomarkers identified for the discrimination SCOPD and AECOPD in this work, except for fucose, exceeded the AUC of the CRP biomarker (Table S9). Notably, the AUC of the eight metabolic biomarker panel is greater than 0.9, much better than for the CRP biomarker. Besides, the clinical diagnostic methods for discriminating SCOPD from AECOPD are time-consuming (the detection of multiple indicators, including respiratory rate, heart rate, oxygen saturation level, C-CRP levels, etc., will take several hours to days).^77^ In contrast, our nanoplatform offers a fast (within 1 h after getting the blood) tool for the diagnosis of AECOPD, facilitating the early diagnosis of AECOPD.

Biomarkers can not only be used for the construction of diagnostic models but also provide available information on the disease’s progress. For example, lactic acid is produced in large amounts in AECOPD patients due to their poor ability to breathe, which leads to the limitation of oxygen and enhancement of glycolysis.^81,82^ A serum uric acid level that increases significantly during hypoxia is associated with a higher risk of AECOPD and hospitalizations.^83^ Malondialdehyde concentrations are also elevated in AECOPD, requiring hospitalizations compared with stable COPD.^84^ Creatine and dimethylglycine commonly showed a decrease in blood due to the oxidation of creatine oxidation for AECOPD patients.^85,86^ 3-Hydroxybutyric acid was increased, which may be attributed to the energy metabolism shift from carbohydrates to lipid utilization and amino acid metabolism in AECOPD.^87^ Threonine was found to be a potential biomarker for AECOPD diagnosis.^88,89^ Fucose may be the degradation product of the fucosylation glycoprotein, which was a biomarker of COPD.^90^ These metabolic biomarkers will provide powerful tools for the assessment and management of COPD. Furthermore, eight metabolic biomarkers were used with pathway enrichment analysis to explore biological relevance. The results demonstrated that the glycine, serine, and threonine metabolism were the most significantly altered metabolic pathways associated with COPD exacerbation (Figure S25, Table S10). The reason for amino acid metabolism dysfunction may arise from systemic inflammation and impaired energy metabolism in skeletal muscles.^86,91^

## Conclusions

We constructed mesoporous PdPt alloys to reveal unique metabolic signatures for the diagnosis of COPD and exacerbations. The optimized PdPt alloys exhibited superior performance in metabolite detection, attributed to the enhanced electric field, robust photothermal conversion, and strengthened photocurrent response. The platform achieved precise diagnosis of COPD and exacerbations, with simple sample pretreatment, minimal sample consumption, and high speed. It is noteworthy that we screened distinct metabolic biomarkers for the diagnosis of COPD exacerbations, revealing the metabolic signatures in COPD progress. Although there are still limitations in the study, such as the fact that more samples should be collected from the multicenter to further validate the reliability of our platform and further study needs to be conducted to explore the mechanism of how metabolic biomarkers contribute to the AECOPD onset and progression, we believe that our platform would provide a noninvasive and robust tool to advance metabolic analysis for disease diagnosis and prognosis.

## Supporting Information Available

Detailed chemicals, reagents, experimental procedures (preparation of mesoporous PdPt nanoparticles, material characterization, theoretical simulation, study cohort, LDI MS detection, machine-learning analysis, FT-ICR-MS analysis, UHPLC-MS analysis, statistical analysis), and supplementary figures and tables (PDF)
