# Supplementary material for: Plasmonic Alloys Enhanced Metabolic Fingerprints for the Diagnosis of COPD and Exacerbations
Source: ACS Cent Sci. 2024 Jan 27;10(2):331–43. doi: 10.1021/acscentsci.3c01201 (PMC10906255; doi:10.1021/acscentsci.3c01201)
Supplement: Supplementary file 1 — oc3c01201_si_001.pdf [file oc3c01201_si_001.pdf]

Supporting Information

Plasmonic alloys enhanced metabolic fingerprints for diagnosis of COPD and exacerbations

Authors

Haiyang Su[1†], Yuanlin Song[2,3,4†], Shouzhi Yang[1], Ziyue Zhang[1], Yao Shen[5], Lan Yu[6,7,8], Shujing Chen[2,3], Lei Gao[2,3], Cuicui Chen[2,3], Dongni Hou[2,3], Xinping Wei[9], Xuedong Ma[9], Pengyu Huang[9], Dejun Sun[7,10], Jian Zhou[2,3,4]*, Kun Qian[1, 11]*

Affiliations

[1]State Key Laboratory of Systems Medicine for Cancer, School of Biomedical Engineering, Institute of Medical Robotics and Med-X Research Institute, Shanghai Jiao Tong University, Shanghai, 200030, P. R. China.

[2]Department of Pulmonary and Critical Care Medicine, Shanghai Respiratory Research Institute, Zhongshan Hospital, Fudan University, Shanghai, 200032, P. R. China.

[3]Shanghai Key Laboratory of Lung Inflammation and Injury, 180 Fenglin Road, Shanghai, 200032, P. R. China.

[4]Center of Emergency and Critical Medicine, Jinshan Hospital of Fudan University, Shanghai, 201508, P. R. China.

[5]Department of Respiratory and Critical Care Medicine, Shanghai Pudong Hospital, Fudan University, Shanghai, 201399, P. R. China.

[6]Clinical Medical Research Center, Inner Mongolia People's Hospital, Hohhot, 010017, Inner Mongolia, P. R. China.

[7]Inner Mongolia Key Laboratory of Gene Regulation of The Metabolic Disease, Inner Mongolian People's Hospital, Hohhot, 010017, Inner Mongolia, P. R. China.

[8]Inner Mongolia Academy of Medical Sciences, Inner Mongolian People's Hospital, Hohhot, 010017, Inner Mongolia, P. R. China.

[9]Shanghai Minhang District Gumei Community Health Center affiliated to Fudan University, Shanghai, 201102, P. R. China.

[10]Department of Respiratory and Critical Care Medicine, Inner Mongolia People's Hospital, Hohhot, 010017, P. R. China.

[11]Shanghai Key Laboratory of Gynecologic Oncology, Renji Hospital, School of Medicine, Shanghai Jiao Tong University, Shanghai, 200127, P. R. China.

*Corresponding author: Kun Qian (k.qian@sjtu.edu.cn), Jian Zhou (zhou.jian@fudan.edu.cn)

†These authors contributed equally to this work.

This file included:

1. Chemicals and reagents
2. Experimental procedures
3. Figure S1-S25 and Table S1-S10
4. References

1. Chemicals and reagents

Chloroplatinic acid hexahydrate ($H_2PtCl_6·6H_2O$, 37%), hydrochloric acid (HCl, 36%~38%), sodium chloride (99.5%), methanol (99.8%), ethanol absolute (99.7%) and acetonitrile (99.5%) were ordered from Sinopharm Chemical Reagent Beijing Co., Ltd (Beijing, China). Pluronic F127, phenylalanine (98%), glucose (99.5%), proline (99.0%), asparagine (98.0%), arginine (99.5%) and sucrose (99.0%), alanine (98.0%), serine (99.0%), valine (98.0%), lysine (98.0%), threonine (98.0%), methionine (98.0%), histidine (99.0%), L-tryptophan (99.0%), isoleucine, aspartic acid (99.0%), glutamine (99.0%), maltose (99.0%), arabinose (99.0%), lactose (99.0%), melamine (99.5%), niacinamide (99.5%), uracil (99.0%), adenine, guanine (99.0%), succinic acid (99.0%), adipic acid (99.5%), 2,5-dihydroxybenzoic acid (DHB), alpha-cyano-4-hydroxycinnamic acid (CHCA) and bovine serum albumin (BSA, 98%) were purchased from Sigma-Aldrich (St. Louis, MO, USA). Sodium tetrachloropalladate (Na_2PdCl4, 98%) and ascorbic acid (99%) were ordered from Aladdin Reagent Co., Ltd (Shanghai, China). Trifluoroacetic acid (TFA, 99.5%) was ordered from Macklin Biochemical Co., Ltd. (Shanghai, China).

All aqueous solutions throughout the experiments were prepared with deionized water (18.2 MΩ·cm, Milli-Q, Millipore, GmbH).

2. Experimental procedures

Preparation of mesoporous PdPt nanoparticles

Mesoporous PdPt alloys were synthesized via a modified surfactant-directed method[1]. Briefly, 3 mL of Na_2PdCl_4 solution (20 mM), 15 mL of $H_2PtCl_6·6H_2O$ (20 mM), HCl (with concentrations of 3 M (250 μL), 6.0 M (250 μL), 12.0 M (250 μL), and 12 M (500 μL), respectively and the corresponding PdPt alloys were denoted as PdPt-1/2/3/4), and 300 mg of Pluronic F127 were mixed to a homogeneous solution, following the addition of 15 mL of ascorbic acid solution (0.1 M). After that, the mixed solution was continuously sonicated in a water bath at 45℃ for 4 hours to obtain different particle sizes of PdPt micelles. The products were collected by centrifugation at 10,000 rpm for 10 min and the residual Pluronic F127 template was removed by three consecutive washing/centrifugation cycles with ethanol and water. Finally, the porous PdPt nanoparticles were dried at 50℃ for 12 hours before use.

Material characterization

Scanning electron microscope (SEM) images and energy-dispersive X-ray (EDX) spectra were conducted by using an S-4800 (Hitachi, Japan), by dropping ~2 μL of water suspension of materials on the aluminum foil. Transmission electron microscope (TEM) images, high-resolution TEM (HRTEM) images, selected-area electron diffraction (SAED) pattern, elemental mapping images, and line-scan EDX analysis were carried out using a JEM-2100F instrument (JEOL, Japan), by depositing the diluted colloidal suspension on a copper grid. Low-angle XRD pattern was recorded on a Pert3 Powder5 (PANalytical, Holland) with Cu Kα radiation (λ = 0.154 nm). Wide-angle powder X-ray diffraction (XRD) pattern was performed on a D8 Advanced X-Ray Diffractometer (Bruker, Germany) with Cu Kα radiation. X-ray photoelectron spectroscopy (XPS) was performed with the Thermo Scientific K-Alpha XPS system. Zeta potential measurements were recorded on a Nano-ZS90 instrument (Malvern, Worcestershire, UK) in water at room temperature. Ultraviolet-visible (UV-Vis) absorption spectra were measured by a UV1900 spectrophotometer (AuCy, China). The photocurrents were recorded in a standard three-electrode system with platinum wire as a counter electrode, Ag/AgCl as a reference electrode, and 0.5 M Na_2SO_4 as electrolyte solution in a CHI 660E electrochemistry workstation (Beijing Chinese science days Technology Co., Ltd). The nitrogen adsorption-desorption analysis was performed by Autosorb IQ-MP.

Theoretical simulation

To study the differences in the optical response of the synthesized nanoparticles, especially the influence of the porous structure and particle size, the finite element method (FEM) provided by the Wave Optical Module of COMSOL Multiphysics is used to solve the Holmholtz equation about time-harmonic electric field (E):

$$\nabla\times\left(\mu_r^{-1}\nabla\times E\right)-k_0^2\varepsilon_r E=0 \qquad (1)$$

Where μ_r=1 was the relative permeability, k_0 was the wave vector, $\varepsilon_r=(n+ik)^2$ was the relative permittivity[2]. The refractive index of Pt and Pd is modeled using the experimental data of Werner et al. with linear interpolation[3]. The particle size, pore size, and composition of nanoparticles refer to SEM, TEM and EDS results. The refractive index of the air is set as 1. The wavelength of incident light was set at 355 nm matched with the wavelength of laser source equipped in laser desorption/ionization mass spectrometry (LDI MS). The calculated region is surrounded by a spherical PML layer.

The relationship between electric field intensity (I) and electric field (E) is:

$$I = |E|^2, \qquad (2)$$

The equation for calculating the dissipated power density (Q) is:

$$Q = \frac{1}{2} Re(J^* \cdot E), \qquad (3)$$

where J is the current density.

The temperature caused by photothermal conversion was calculated based on the equation:

$$\rho C_p \frac{\partial T}{\partial t} + (-k\nabla T) = Q$$

where ρ is the density, C_p is the heat capacity at constant pressure, k is the thermal conductivity, ∇T is the temperature variation, and t is the time. The initial temperature was set at 293.15 K.

Study cohort

In this study, 431 individuals were recruited from Zhongshan Hospital, Fudan University and Shanghai Pudong Hospital, Fudan University, including 185 healthy controls and 246 chronic obstructive pulmonary disease (COPD) patients (122 stable COPD (SCOPD) and 124 acute exacerbation of COPD (AECOPD) individuals). The healthy controls were verified by spirometry with normal lung function, and all patients were diagnosed with persistent airflow limitation based on the clinic criteria[4]. The study was approved by the Ethics Committee of Zhongshan Hospital (approval number B2020-428R) and Shanghai Pudong Hospital (approval number 2021-SKWXZ-01). All participants gave their informed written consent before entering the study, and kept an overnight fasting (more than 8 hours) before the plasma collection. The peripheral blood of each participant was collected with centrifugation at 3000 × g for 10 min to obtain plasma, and stored at -80° C before further analysis. All experiments were performed following institutional guidelines, in compliance with relevant laws.

For the distinction between healthy controls and COPD patients, we randomly assigned 431 subjects to a discovery cohort (143 healthy controls and 166 patients) and an independent validation cohort (42 healthy controls and 80 patients). For the distinction between healthy controls and SCOPD patients, we also randomly grouped the 273 subjects into a discovery cohort (105 healthy controls and 103 SCOPD patients) and an independent validation cohort (46 healthy controls and 19 SCOPD patients). For the distinction between healthy controls and

AECOPD patients, we randomly assigned 275 subjects to a discovery cohort (105 healthy controls and 102 AECOPD patients) and an independent validation cohort (46 healthy controls and 22 AECOPD patients). Moreover, for the distinction between SCOPD and AECOPD, we also randomly grouped the 246 subjects into (1) a discovery cohort of 103 SCOPD patients and 102 AECOPD patients, and an independent validation cohort of 19 SCOPD patients and 22 AECOPD patients. No significant differences existed in age and gender in all discovery cohorts.

LDI MS detection

For the detection of standard small metabolites, proline (Pro), asparagine (Asn), phenylalanine (Phe), arginine (Arg), glucose (Glc), and sucrose (Suc) were co-dissolved in deionized water. These molecules were also mixed with salts (NaCl, 0.5 M) and proteins (BSA, 5 mg mL^{-1}) to explore the detection efficiency of PdPt alloys in high concentrations of salt and protein.

For the detection of metabolites in plasma, plasma was first pretreated with organic solvents to precipitate some protein. Briefly, 10 μL of plasma was mixed with 10 μL of organic solvents (methanol/acetonitrile, v/v=1:1), and incubated for 10 minutes on an orbital shaker. After that, the sample was centrifuged at 10,000 rpm for 10 minutes, removing the sediment and retaining the supernatant for further analysis.

In a typical LDI MS experiment, 1 μL of analyte solution (standard small metabolites or plasma samples) was spotted on the polish plate and dried in air at room temperature, followed by adding 1 μL of matrix suspension and dried for LDI MS analysis. For PdPt alloys, they were dispersed in water at a concentration of 1 mg mL^{-1} for use as a matrix. For the organic matrix (CHCA and DHB), they were dissolved in 0.1% TFA solution (water/acetonitrile=7/3, v/v) to prepare a saturated and 10 mg/mL solution, respectively. Mass spectra were recorded on an AutoFlex TOF/TOF mass spectrometer (Bruker, Germany) equipped with a Nd:YAG laser (2 kHz, 355 nm) at a positive model. The pulse frequency and number of laser shots for each detection were set as 1000 Hz and 2000, respectively. The acceleration voltage was set as 20 kV and the delay time was optimized to 200 ns.

Machine-learning analysis

For a typical machine-learning classification, five mass spectra obtained for each sample were used to build molecular databases. The raw mass spectra data was pre-processed with

baseline correction, peak detection, extraction, alignment, normalization, and standardization in MATLAB (R2016a, The MathWorks, Natick, MA). Then, the MS data matrix with sample label ('0' for healthy control and '1' for COPD patient) was analyzed by sparse learning (Elastic Net), using a "home-built" code by MATLAB. The classification was set with 5-fold cross-validation to estimate the performance of the predictor, with 20 rounds for each fold yielding 100 models in total. The performance of the classifier was evaluated by the area under the curve (AUC) of the receiver operation curve (ROC), calculating the proportions of concordant pairs among all pairs of observations, with 1 indicating perfect prediction accuracy. To identify the metabolic signature that contributed the most to the classifier, we chose m/z features according to (1) the model selected frequency with repeat occurrence ≥ 90% in 100 models; (2) $p < 0.05$ according to two-sided t-test; (3) AUC > 0.7 of a single feature, and (4) the abundance of the feature is over 500. The potential metabolic biomarkers were identified based on accurate mass from online database (HMDB) and the Fourier Transform-Ion Cyclotron Resonance-Mass Spectrometry (FT-ICR-MS) results.

FT-ICR-MS analysis

To further validate the screening metabolic features, the accurate MS for potential biomarkers was measured on the Fourier Transform-Ion Cyclotron Resonance-Mass Spectrometry (FT-ICR-MS). For each group (healthy control, SCOPD and AECOPD), we randomly selected 50 plasma samples from 50 different subjects and took 10 μL plasma from each sample to mix them. Then 10 μL of mixed plasma was mixed with 10 μL of organic solvents (methanol/acetonitrile, v/v=1:1), and incubated for 10 minutes on an orbital shaker. After that, the sample was centrifuged at 10,000 rpm for 10 minutes, removing the sediment and retaining the supernatant. Then, 1 μL of plasma extracts was spotted on the polish plate and dried in air at room temperature, followed by adding 1 μL of matrix suspension (PdPt alloys with 1 mg mL^{-1}) and dried for FT-ICR-MS analysis.

UHPLC-MS analysis

To identify the screening metabolic biomarkers, the plasma was further performed for ultra-performance liquid chromatography-MS (UHPLC-MS) analysis. For each group (healthy control, SCOPD and AECOPD), we also randomly selected 50 plasma samples from 50 different subjects and took 10 μL plasma from each sample to mix them. For each group, we

took 100 μL mixed plasma for UHPLC-MS analysis. The sample was treated with 400 μL solvents of methanol:acetonitrile (1:1, v/v), full vortex oscillation for 30 s. The solution was incubated at -20℃ for 2 h to allow for protein precipitation. After that, the supernatant was collected by centrifugation at 12,000 rpm and 4℃ for 15 min. The supernatant was dried in a vacuum centrifuge for 4 hours, following re-dissolved in 100 μL of solvents (methanol/water, v/v=3:7) and centrifuged at 12,000 rpm and 4°C for 15 min to remove insoluble debris. The supernatant was transferred to vials for further analysis. Chromatographic analysis was performed on a Vanquish UHPLC/Q Exactive plus (Thermo Scientific, USA) with an ACQUITY UPLC HSS T3 column (100×2.1 mm, 1.7 μm, Waters). The mobile phases are A (water with 0.1% formic acid) and B (acetonitrile with 0.1% formic acid), with the gradient of A/B 99/1~0/100 in 12min and holding in 0/100 for 12-13min.

Data were acquired by software Xcalibur 3.0 (Thermofisher) and processed by Progenesis QI v2.3 data analysis software (Waters, UK) for peak picking, alignment, and normalization. The metabolites were identified by accurate mass (mass error < 5 ppm), MS/MS spectra and human metabolome database.

Statistical analysis

All the univariate analyses in this work were performed to calculate the *p* value for statistical demonstration, based on the SPSS software (version 24.0, SPSS Inc., Chicago). Power analysis was conducted on the Metaboanalyst 5.0 (https://www.metaboanalyst.ca/). Figures were plotted using GraphPad Prism (GraphPad) and Origin software (OriginLab). Principal component analyses (PCA) were performed using the SIMCA software package (version 14.1, Umetrics, Sweden)[5]. Before analysis, all mass spectra were scaled to Pareto (par) by dividing variables using the square root of the standard deviation when centering was completed.

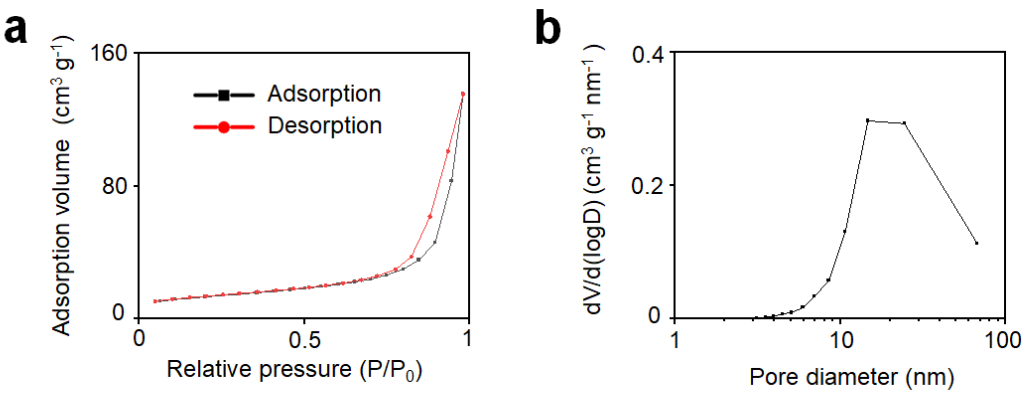

Figure S1. Nitrogen adsorption analysis of PdPt alloys. The nitrogen adsorption-desorption isotherm (a) and pore size distribution (b) of PdPt alloys. The specific surface area was calculated by the Brunauer-Emmett-Teller (BET) model to be ~23.29 m^2 g^{-1}.

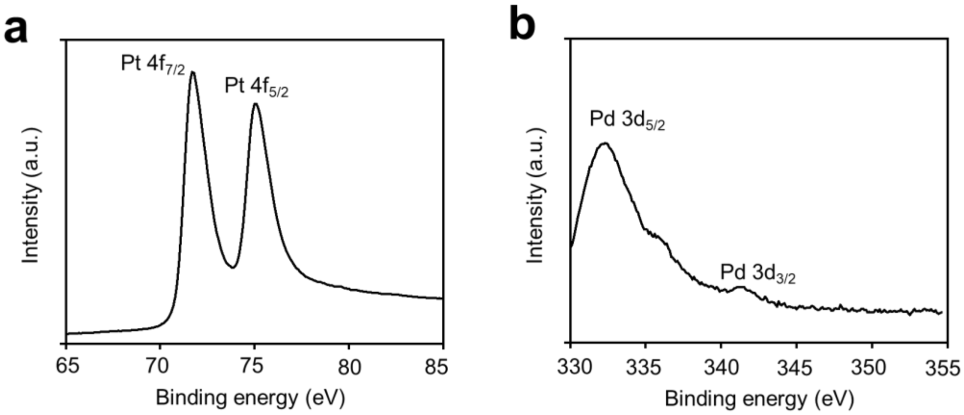

Figure S2. X-ray photoelectronic spectroscopy (XPS) spectra of porous PdPt alloys. XPS spectra of porous PdPt alloys on (a) Pt 4*f* and (b) Pd 3*d*.

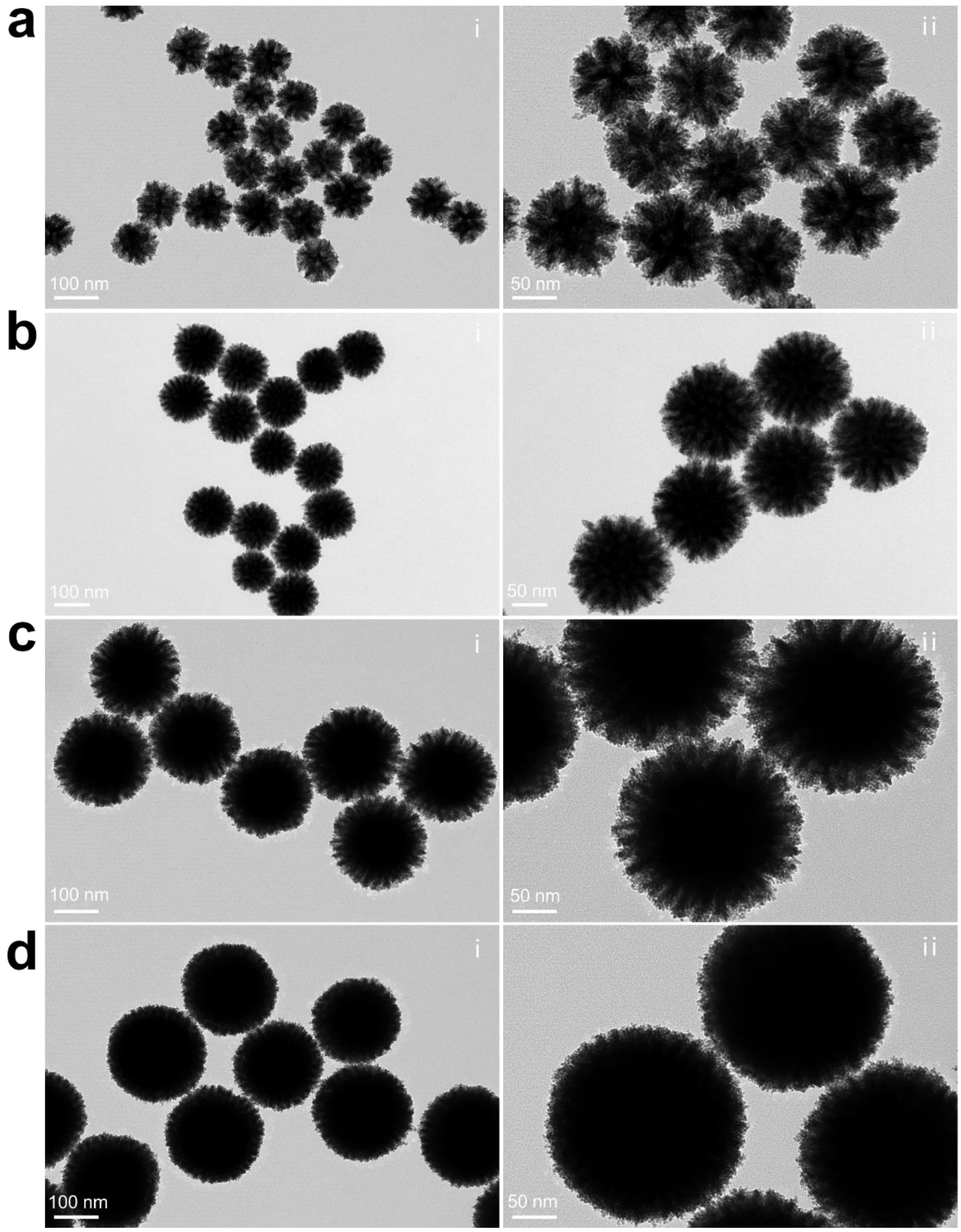

Figure S3. Transmission electron microscopy (TEM) images of porous PdPt alloys with different particle sizes. TEM images of (a) PdPt-1, (b) PdPt-2, (c) PdPt-3 and (d) PdPt-4 alloys. Scale bar: (i) 100 nm and (ii) 50 nm.

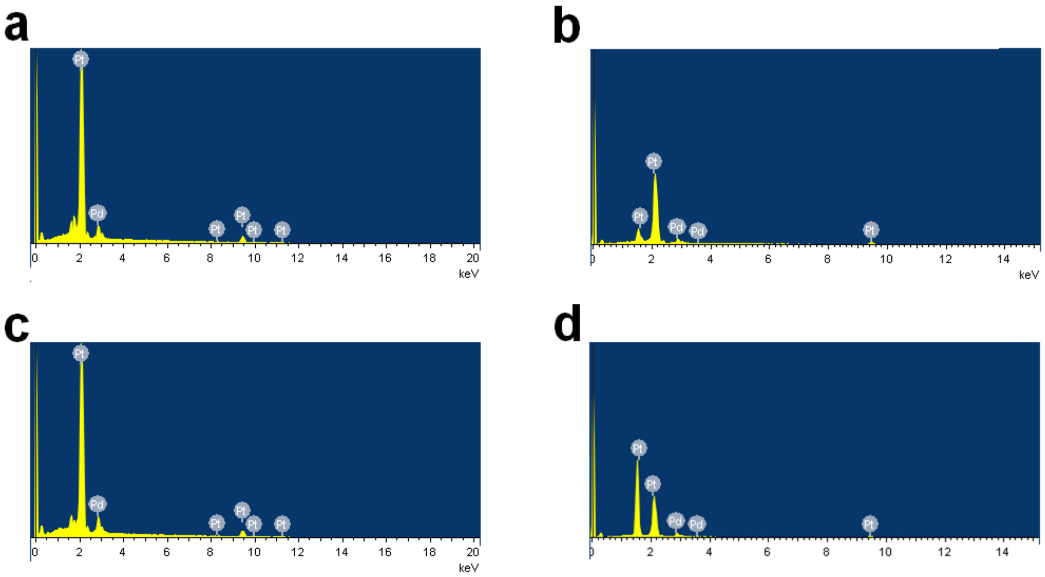

Figure S4. Typical energy-dispersive X-ray (EDX) spectra of porous PdPt alloys. EDX spectra of (a) PdPt-1, (b) PdPt-2, (c) PdPt-3, and (d) PdPt-4 alloys.

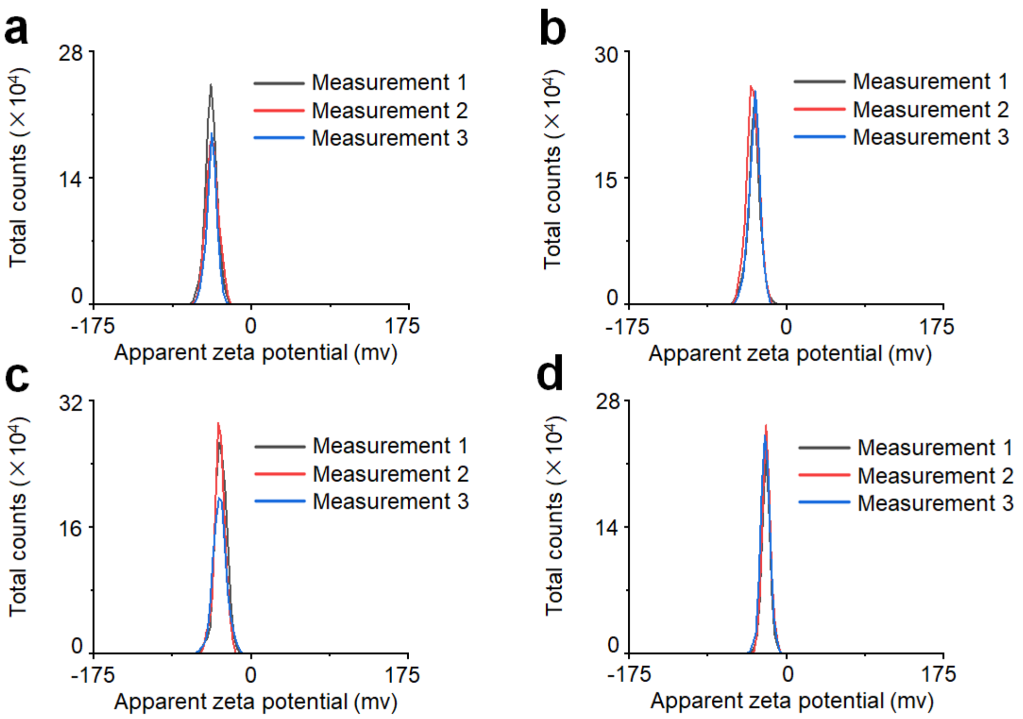

Figure S5. Typical zeta potential distributions of porous PdPt alloys. Zeta potential of (a) PdPt-1, (b) PdPt-2, (c) PdPt-3, and (d) PdPt-4 alloys with three independent measurements, respectively.

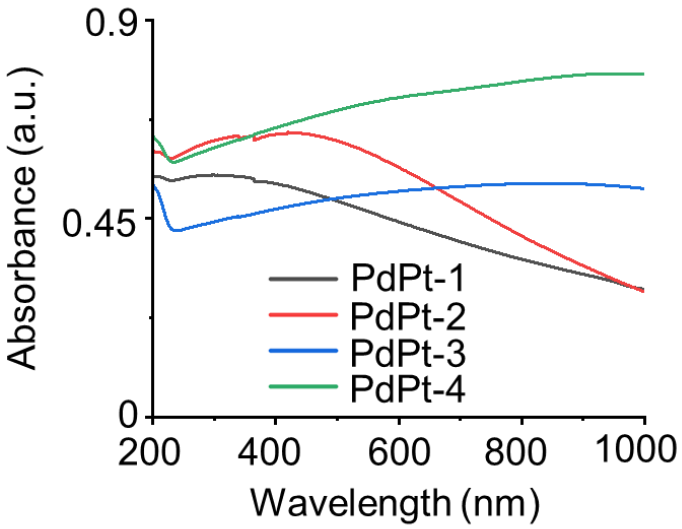

Figure S6. Light absorption of PdPt nanomaterials. Typical ultraviolet-visible (UV-Vis) spectra of PdPt-1/2/3/4 alloys.

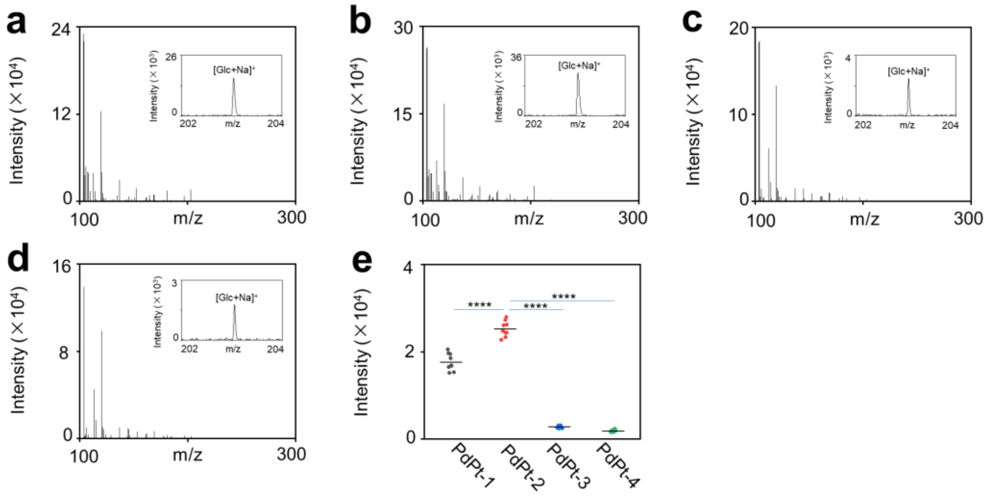

Figure S7. LDI MS detection of plasma extracts by different alloys. Typical mass spectra for 0.5 μL of plasma extracts analyzed by (a) PdPt-1, (b) PdPt-2, (c) PdPt-3, and (d) PdPt-4 alloys assisted LDI MS. The insets were sodium-adducted peaks of typical glucose (Glc) metabolite. (e) Mean intensities of glucose signals were obtained using different alloys as matrices. For each metabolite, 9 replicates were conducted. **** represented $p < 0.0001$.

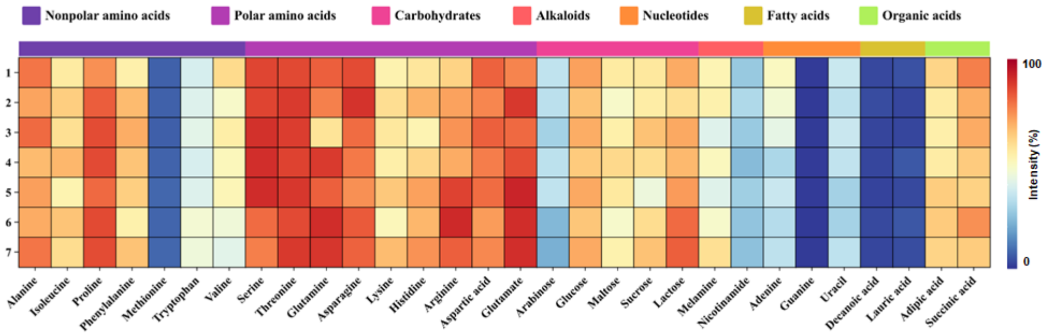

Figure S8. Preference for desorption/ionization of metabolites. All the metabolites including nonpolar amino acids (isoleucine, alanine, proline, phenylalanine, methionine, tryptophan and valine), polar amino acids (serine, threonine, glutamine, asparagine, lysine, histidine, arginine, aspartic acid and glutamate), carbohydrates (arabinose, glucose, maltose, sucrose and lactose), alkaloids (melamine and nicotinamide), nucleotides (adenine, guanine and uracil), fatty acids (decanoic acid and lauric acid) and organic acids (adipic acid and succinic acid) were prepared with a concentration of 1 mg mL^{-1}. The mass spectra were collected in positive ion mode, using PdPt-2 alloys as the matrix. For each metabolite, 7 mass spectra were collected for the heat map.

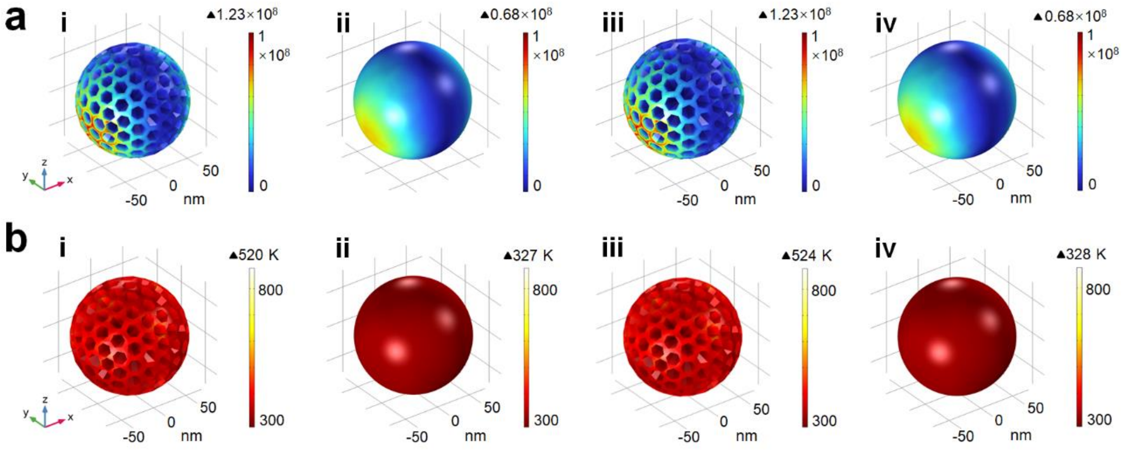

Figure S9. Three-dimensional distributions of electric field and thermal field. Contour plots of (a) electric field amplitudes and (b) thermal field distribution displayed on color scale for (i) porous Pd, (ii) nonporous Pd, (iii) porous Pt, and (iv) nonporous Pt, for 355 nm laser beam polarized along Y-axis. Laser light was injected along Z-axis. Both the electric field amplitudes and thermal field distribution were calculated by the finite element method.

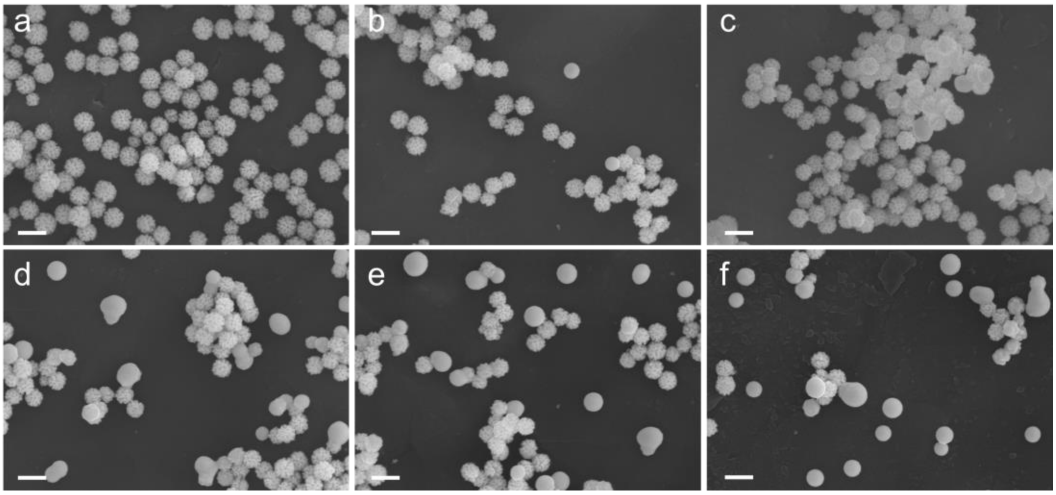

Figure S10. Laser ablation of PdPt-2 alloys. Scanning electron microscopy (SEM) images of PdPt-2 alloys (a) before and after irradiation by 10,000 laser shots at laser fluence of (b) 20%, (c) 40%, (d) 60%, (e) 80%, and (f) 100%. The scale bars were 200 nm.

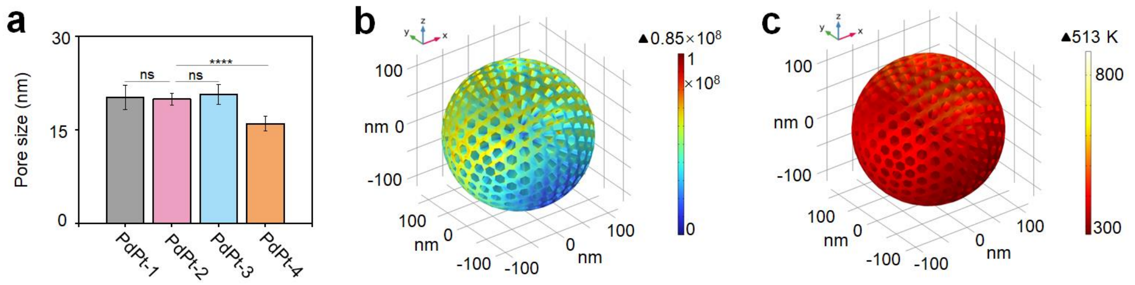

Figure S11. Pore size and theoretical simulation of different PdPt alloys. (a) Comparison of pore size of PdPt-1/2/3/4 alloys. Error bars represent mean ± SD (calculation of 20 pore sizes for each alloy). The average pore sizes of PdPt-1/2/3/4 were about 20.2 nm, 19.9 nm, 20.7 nm and 16 nm, respectively, calculated based on the SEM images (Figure 3d). **** represented $p < 0.0001$. Contour plots of (b) electric field amplitudes and (c) thermal field distribution displayed on the color scale for PdPt-4 alloys with a pore size of 20 nm, for 355 nm laser beam polarized along Y-axis. Laser light was injected along Z-axis.

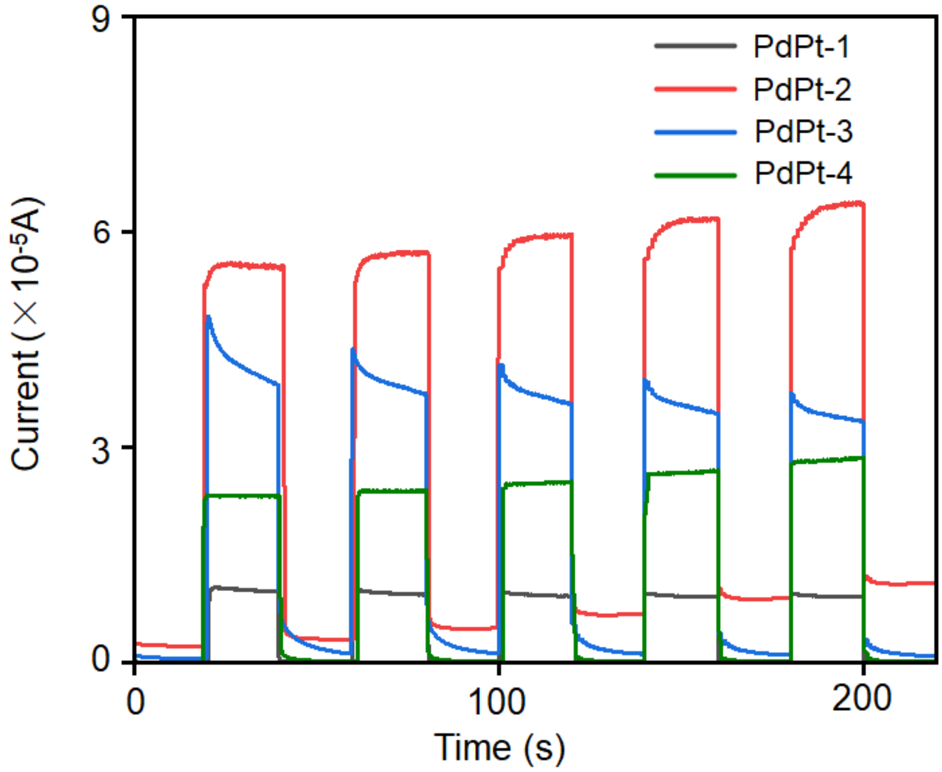

Figure S12. Photocurrent response of PdPt alloys. Typical photocurrent response spectra of PdPt-1/2/3/4 alloys.

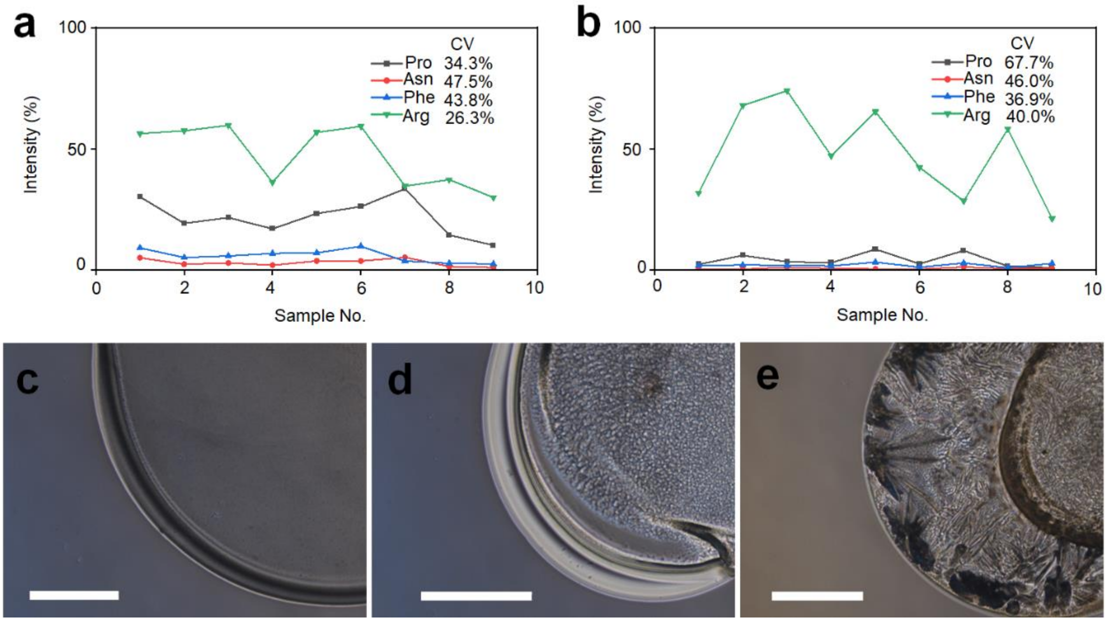

Figure S13. Intensity coefficient of variations (CVs) for metabolites and matrix-analyte co-crystals by different matrix assisted LDI MS. Coefficient of variances (CVs) distribution of different metabolites (proline (Pro), asparagine (Asn), phenylalanine (Phe), arginine (Arg), glucose (Glc) and sucrose (Suc)) detected by (a) CHCA and (b) DHB assisted LDI MS. We can't observe the Glc and Suc adducted peaks when CHCA and DHB as matrix. The results come from 9 independent experiments. Micrographs of matrix-analyte co-crystals using (c) PdPt nanoparticles, (d) CHCA and (e) DHB as matrix. The scale bars were 500 μm.

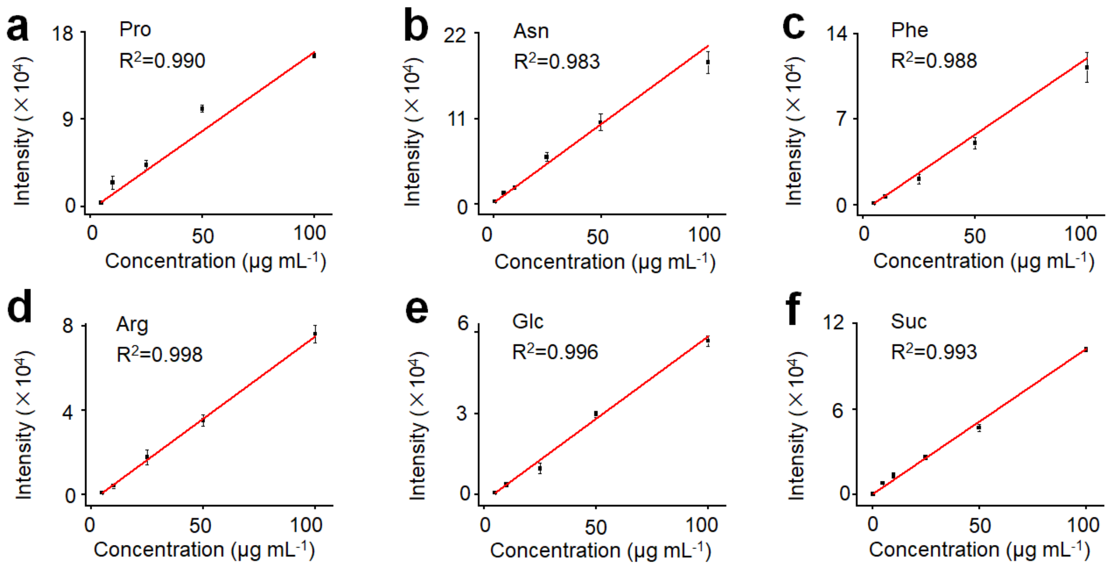

Figure S14. Linear regression of intensity and concentration data of metabolites detected by PdPt alloys. Linear regression of intensity and concentration data of metabolites including (a) proline (Pro), (b) asparagine (Asn), (c) phenylalanine (Phe), (d) arginine (Arg), (e) glucose (Glc), and (f) sucrose (Suc) detected by PdPt alloys. Error bars represent mean ± SD (n=3).

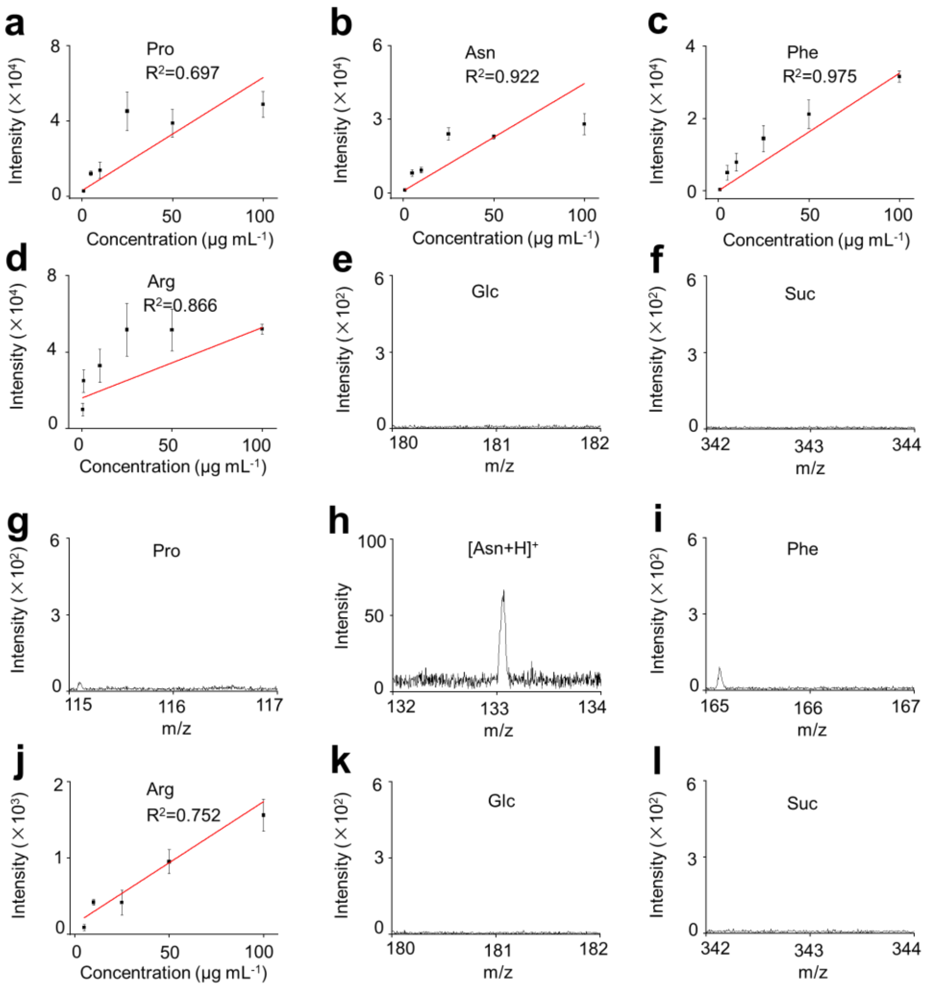

Figure S15. Linear regression and mass spectra of metabolites detected by organic matrix. Linear regression of intensity and concentration data of metabolites including (a) Pro, (b) Asn, (c) Phe, and (d) Arg, and mass spectra of (e) Glc and (f) Suc detected by CHCA. Mass spectra of (g) Pro, (h) Asn, (i) Phe, (k) Glc, and (l) Suc, and (j) linear regression of intensity and concentration data of Arg detected by DHB. The concentration of metabolites for the mass spectra is 100 μg mL^{-1}. Error bars represent mean ± SD (n=3).

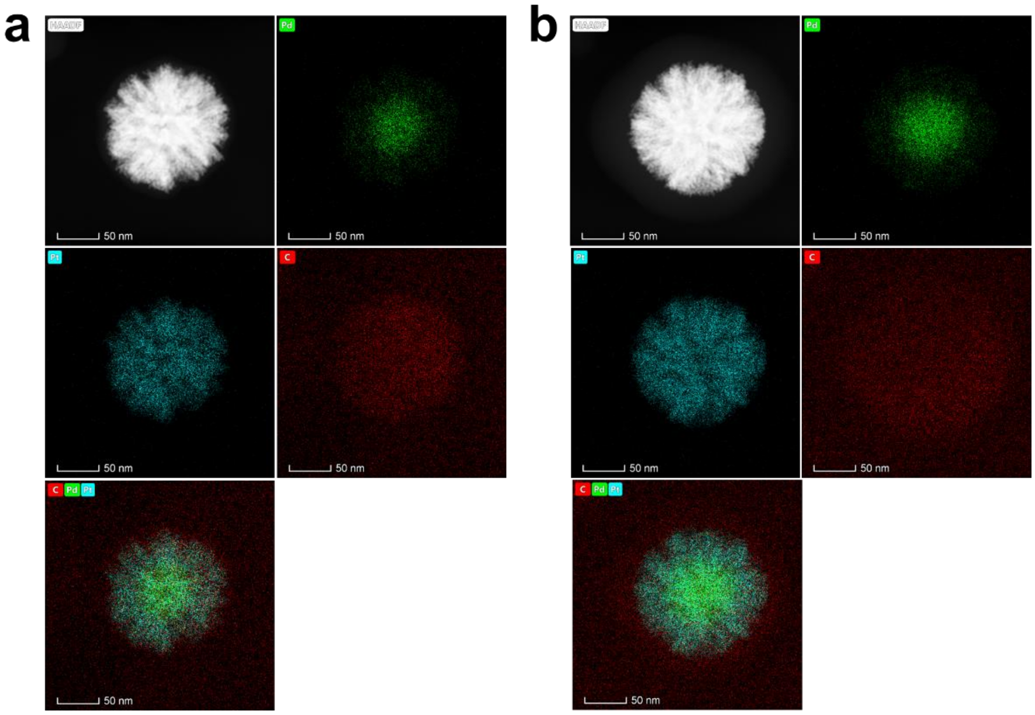

Figure S16. Elemental mapping results. Elemental mappings of Pd, Pt, and C for (a) alloy-glucose and (b) alloy-BSA hybrids. The scale bars were 50 nm.

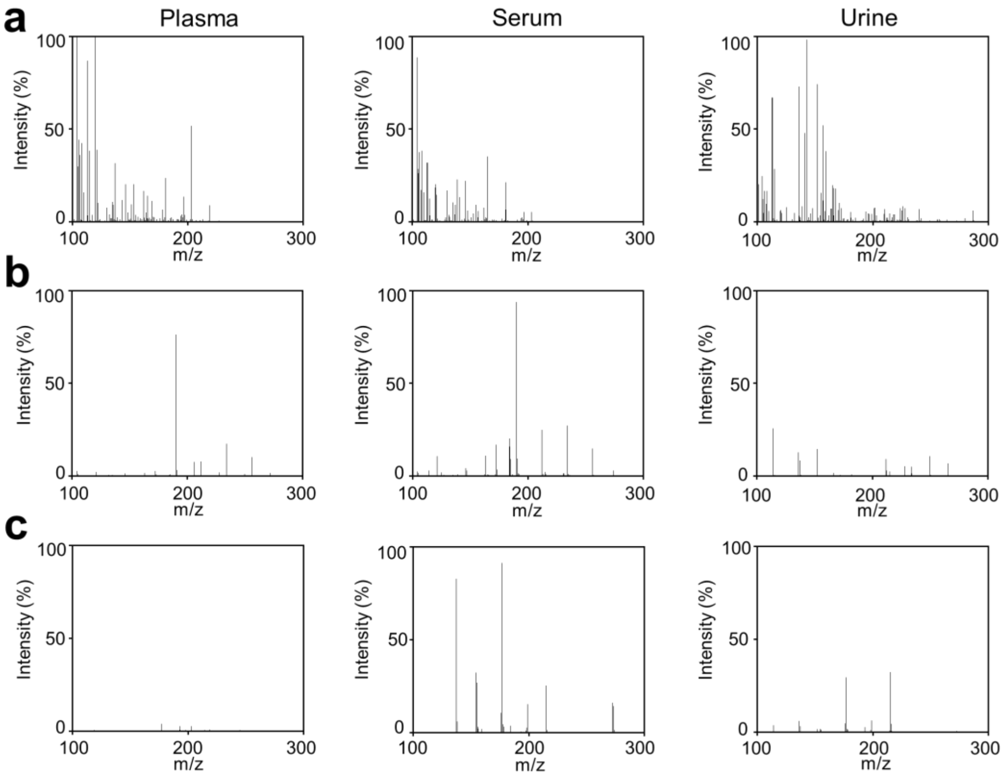

Figure S17. Inorganic matrix and organic matrix for metabolite detection in clinical samples. Mass spectra of metabolites in plasma, serum and urine measured by (a) PdPt alloys, (b) CHCA, and (c) DHB assisted LDI MS.

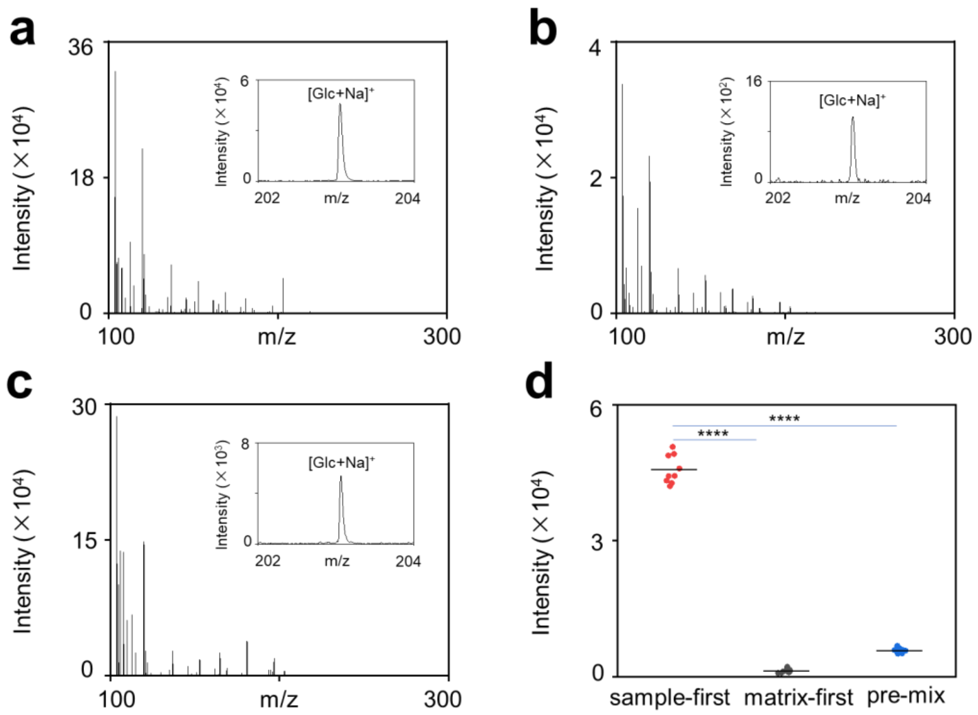

Figure S18. Optimization of sample preparation methods. Typical mass spectra for 0.5 µL of plasma using (a) sample-first method, (b) matrix-first method, and (c) pre-mix method. The insets were sodium-adducted peaks of typical glucose metabolite. (d) Mean intensities of glucose signals were obtained using different sample preparation methods. For each method, 9 replicates were conducted. **** represented $p < 0.0001$.

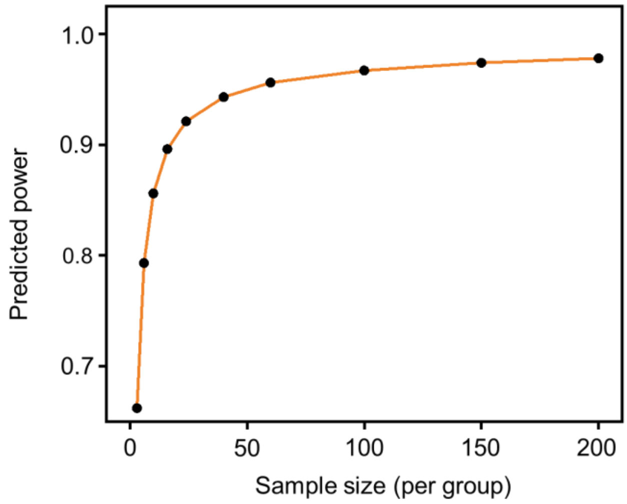

Figure S19. Power analysis. Power analysis based on plasma metabolic fingerprints, including 12 samples (6/6, healthy controls/COPD patients) for calculating the required minimal sample number for machine learning. A power of 0.92 could be obtained with the sample number of 48 (24/24, healthy controls/COPD patients) at a false discovery rate of 0.10.

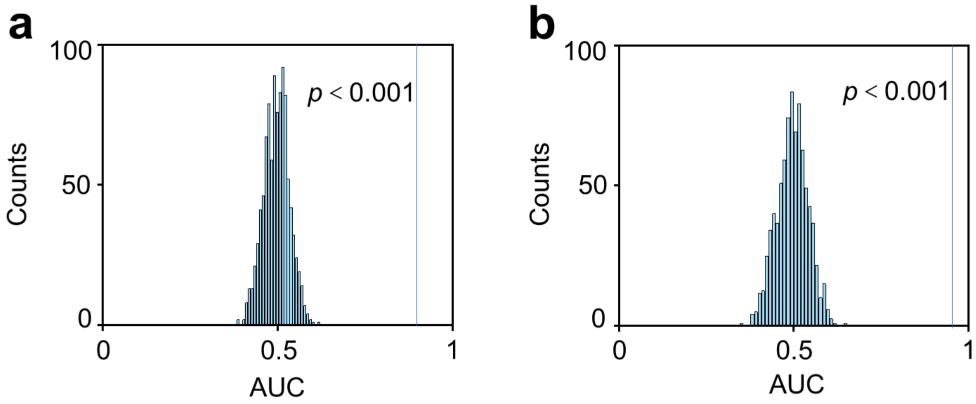

Figure S20. Permutation test in machine learning. The distributions of AUC were calculated by 1000 random permutations. There was no overfitting of the machine learning model as built (p <0.001)

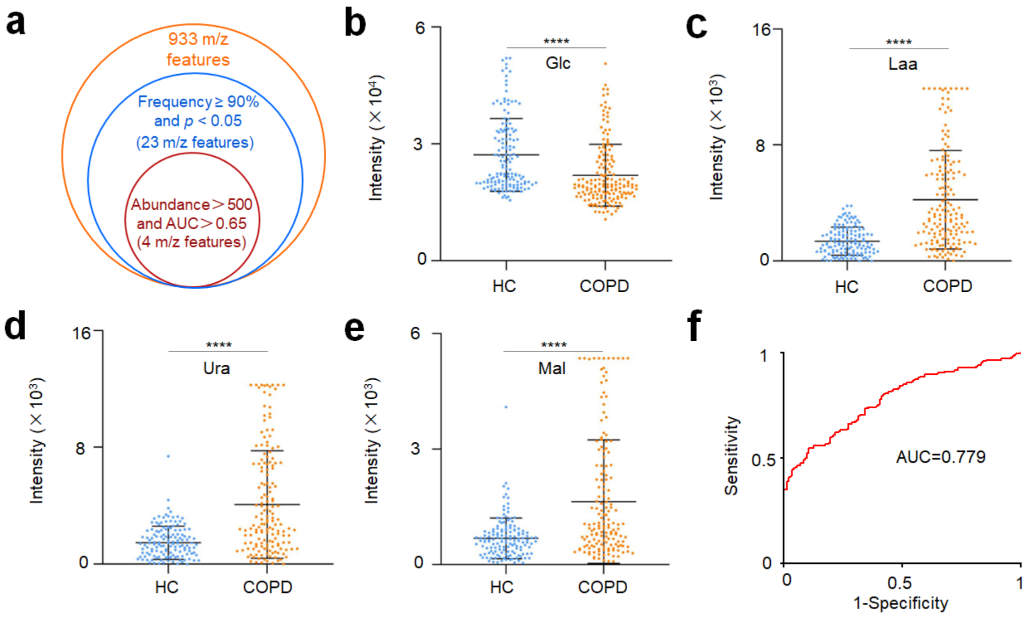

Figure S21. Characterization of significant metabolic biomarkers for COPD diagnosis. (a) Venn diagram of 4 m/z features selected as the metabolic biomarkers based on the frequency ≥ 90%, $p < 0.05$, abundance > 500 and AUC > 0.65. Scatter diagram of 4 significant metabolic biomarkers for COPD diagnosis, including (b) glucose (Glc), (c) lactic acid (Laa), (d) uric acid (Ura) and (e) malondialdehyde (Mal). The **** represented $p < 0.0001$. (f) The ROC curve produced by 4 significant metabolic biomarkers for COPD diagnosis in the discovery cohort.

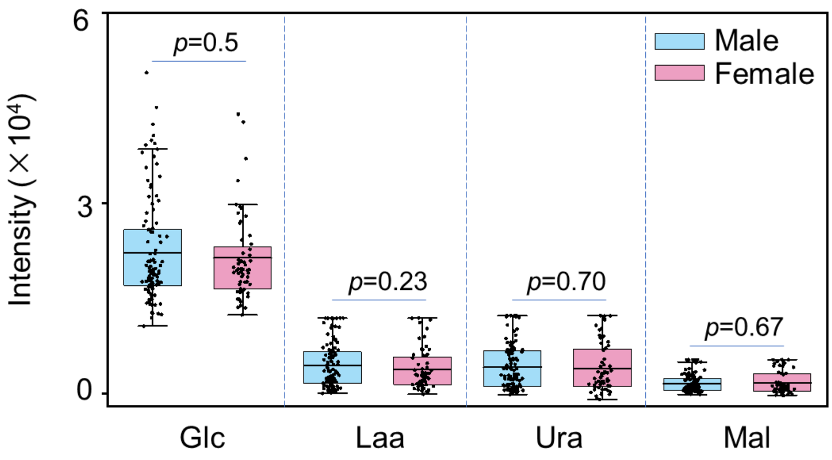

Figure S22. Metabolite difference in males and females. The box diagram demonstrated the expression of 4 metabolites including glucose (Glc), lactic acid (Laa), uric acid (Ura) and malondialdehyde (Mal) in males and females. The samples come from COPD disease group in the discovery cohort, including 106 males and 60 females. The *p* values were calculated by t-test.

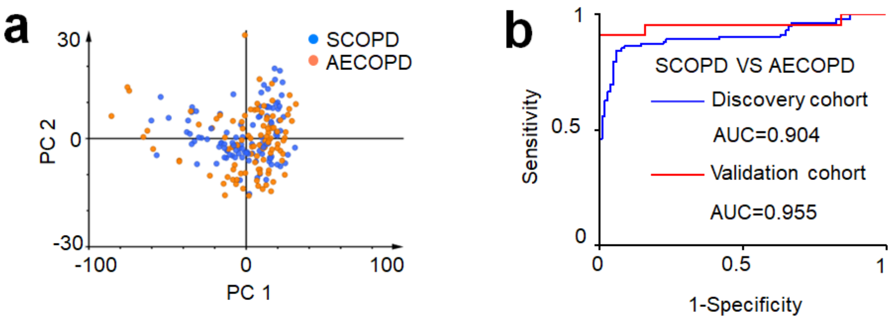

Figure S23. The distinction between SCOPD and AECOPD. (a) PCA analysis based on all 912 m/z metabolic signals extracted in plasma. (b) ROC curves produced by machine learning based on 8 metabolic biomarkers in the discovery cohort and validation cohort.

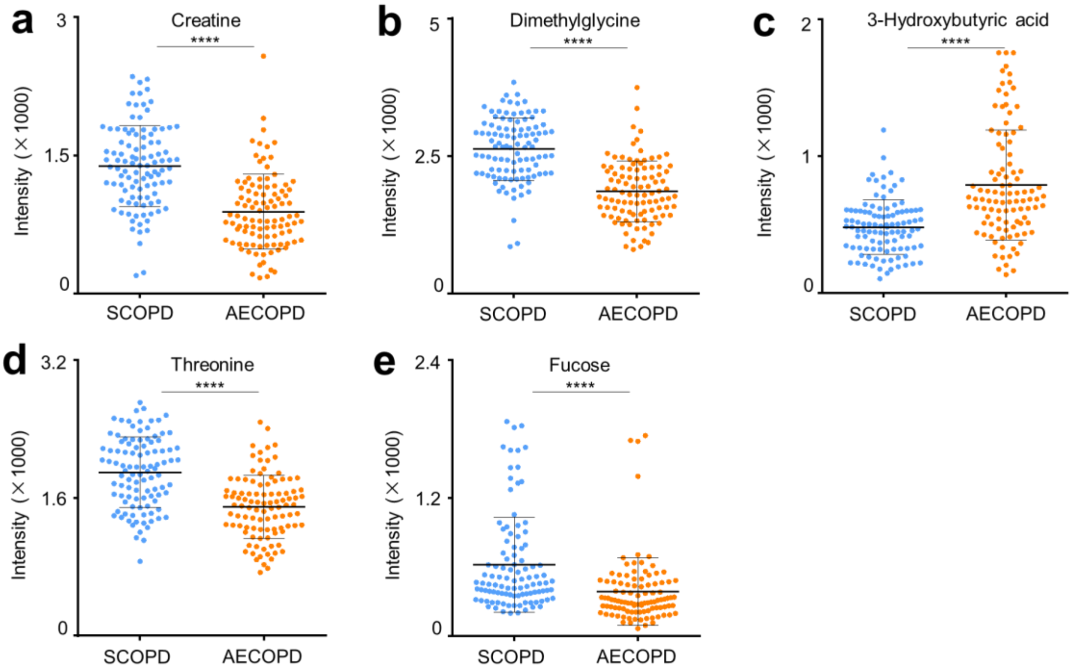

Figure S24. Scatter diagrams of metabolic features between SCOPD and AECOPD. Scatter diagrams of metabolic features, including (a) creatine, (b) dimethylglycine, (c) 3-hydroxybutyric acid, (d) threonine and (e) fucose, were plotted to demonstrate the metabolic difference between SCOPD and AECOPD.

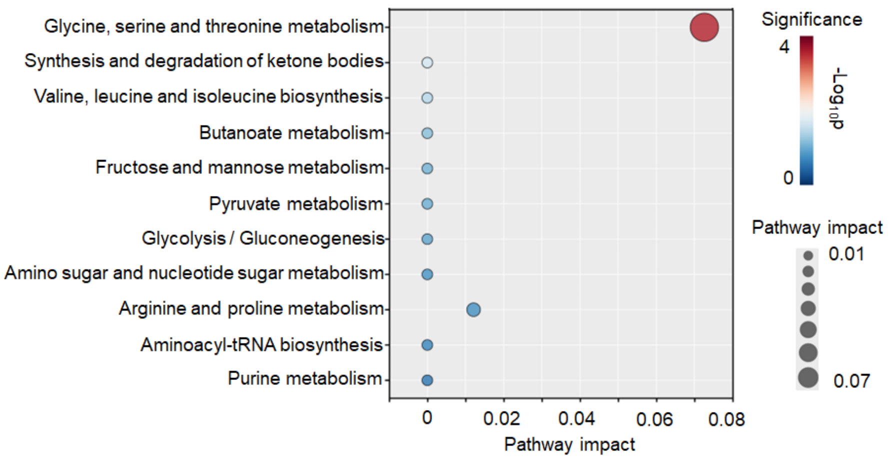

Figure S25. Metabolism pathway enrichment analysis based on 8 metabolic biomarkers for the discrimination AECOPD from COPD. The pathway analysis was based on the Kyoto Encyclopedia of Genes and Genomes (KEGG) pathway and drawn by Chiplot (https://www.chiplot.online/). There were 2 metabolic pathways related to AECOPD with a pathway impact > 0 and hit number (the number of matched metabolites in the pathway) ≥ 1, including 1) glycine, serine and threonine metabolism and 2) arginine and proline metabolism (Table S10).

Table S1. Elementary composition analysis of porous PdPt with different particle size.

Materials	Weight (%)		Atomic (%)	
	Pd	Pt	Pd	Pt
PdPt-1	11.42	88.58	19.12	80.88
PdPt-2	10.35	89.65	17.47	82.53
PdPt-3	12.30	87.70	20.46	79.54
PdPt-4	12.95	87.05	21.43	78.57

Table S2. Apparent zeta potentials of porous PdPt alloys with different particle size.

Materials	Apparent zeta potential[a)]
PdPt-1	-44.07±0.65
PdPt-2	-36.30±0.89
PdPt-3	-34.33±0.72
PdPt-4	-22.77±0.60

[a)] Three independent experiments were performed for each material to calculate the standard deviation (s.d.).

Table S3. Regression equations and limits of detection (LOD) for metabolites detected by different matrices.

Metabolite	Regression equation			R^2			LOD (pmol)		
	PdPt	CHCA	DHB	PdPt	CHCA	DHB	PdPt	CHCA	DHB
Pro	y=1627x-3641	y=600x+3036	/	0.990	0.697	/	25.9	33.2	/
Asn	y=2019x+1274	y=437x+818	/	0.983	0.922	/	3.3	3.8	756.9
Phe	y=1249x-5034	y=325x+15	/	0.988	0.975	/	28.1	13.7	/
Arg	y=784x-3268	y=368x+15983	y=16x+138	0.998	0.866	0.752	28.5	31.5	15.0
Glc	y=610x-2535	/	/	0.996	/	/	26.9	/	/
Suc	y=1020x+19	/	/	0.993	/	/	0.3	/	/

Table S4. Clinical characteristics of healthy controls and COPD patients for discovery and validation cohorts.

Characteristics	Discovery cohort			Validation cohort	
	Healthy control n=143	COPD n=166	*p* value	Healthy control n=42	COPD n=80
Sex			0.43[a)]		
Male	85	106	-	3	75
Female	58	60	-	39	5
Age (median(range))	67 (39-82)	67.5 (37-87)	0.42[b)]	63 (32-85)	81 (69-87)

[a)] *p* value was calculated by $\chi 2$ test; [b)] *p* value was calculated by two-sided Student's t-test.

Table S5. Metabolic biomarkers screened by machine learning and identified through FT-ICR-MS to distinguish COPD from healthy controls.

Metabolite	HMDB ID	Molecular formula	Frequency[a)]	Coefficient[b)]	*p* value[c)]	AUC[d)]
Glucose	HMDB0000122	$C_6H_{12}O_6$	100	-0.23	****	0.69
Lactic acid	HMDB0000190	$C_3H_6O_3$	100	0.87	****	0.78
Malondialdehyde	HMDB0006112	$C_4H_8O_3$	96	0.11	****	0.75
Uric acid	HMDB0000289	$C_5H_4N_4O_3$	100	0.48	****	0.67

[a)] Frequency referring to selection probability by optimized classifier for diagnosis in 100 models; [b)] Coefficient referring to the statistical weight calculated as the sparsity constraints of the diagnostic classifier; [c)] *p* value was acquired for COPD and healthy controls through two-sided Student's t-test (**** $p < 0.0001$); [d)] AUC was acquired by univariate ROC curve analysis for the individual biomarker.

Table S6. Clinical characteristics of healthy controls and stable COPD (SCOPD) patients for discovery and validation cohorts.

Characteristics	Discovery cohort			Validation cohort	
	Healthy control n=105	SCOPD n=103	*p* value	Healthy control n=46	SCOPD n=19
Sex			0.84[a)]		
Male	70	70	-	6	8
Female	35	33	-	40	11
Age (median(range))	69 (42-86)	68 (45-82)	0.52[b)]	61 (32-88)	72 (70-97)

[a)] *p* value was calculated by $\chi 2$ test; [b)] *p* value was calculated by two-sided Student's t-test.

Table S7. Clinical characteristics of healthy controls and exacerbations of COPD (AECOPD) patients for discovery and validation cohorts.

Characteristics	Discovery cohort			Validation cohort	
	Healthy control n=105	AECOPD n=102	*p* value	Healthy control n=46	AECOPD n=22
Sex			0.23[a)]		
Male	77	82	-	6	21
Female	28	20	-	40	1
Age (median(range))	68 (45-82)	70.5 (37-83)	0.79[b)]	61 (32-88)	87 (83-93)

[a)] *p* value was calculated by $\chi 2$ test; [b)] *p* value was calculated by two-sided Student's t-test.

Table S8. Clinical characteristics of SCOPD and AECOPD patients for discovery and validation cohorts.

Characteristics	Discovery cohort			Validation cohort	
	SCOPD n=103	AECOPD n=102	*p* value	SCOPD n=19	AECOPD n=22
Sex			0.21[a)]		
Male	73	82	-	5	21
Female	30	20	-	14	1
Age (median(range))	69 (42-86)	70.5 (37-83)	0.49[b)]	72 (57-97)	87 (83-93)

[a)] *p* value was calculated by $\chi 2$ test; [b)] *p* value was calculated by two-sided Student's t-test.

Table S9. Metabolic biomarkers screened by machine learning and identified through FT-ICR-MS to distinguish AECOPD from COPD.

Metabolite	HMDB ID	Molecular formula	Frequency[a)]	Coefficient[b)]	*p* value[c)]	AUC[d)]
Creatine	HMDB0000064	$C_4H_9N_3O_2$	100	-0.40	****	0.79
Lactic acid	HMDB0000190	$C_3H_6O_3$	100	0.65	****	0.86
Dimethylglycine	HMDB0000092	$C_4H_9NO_2$	100	-0.64	****	0.83
3-Hydroxybutyric acid	HMDB0000011	$C_4H_8O_3$	95	0.12	****	0.75
Uric acid	HMDB0000289	$C_5H_4N_4O_3$	100	0.40	****	0.85
Threonine	HMDB0000167	$C_4H_9NO_3$	100	-0.29	****	0.75
Malondialdehyde	HMDB0006112	$C_3H_4O_2$	100	0.18	****	0.83
Fucose	HMDB0000174	$C_6H_{12}O_5$	97	-0.09	****	0.72

[a)] Frequency referring to selection probability by optimized classifier for diagnosis in 100 models; [b)] Coefficient referring to the statistical weight calculated as the sparsity constraints of the diagnostic classifier; [c)] *p* value was acquired for SCOPD and AECOPD through two-sided Student's t-test (**** $p < 0.0001$); [d)] AUC was acquired by univariate ROC curve analysis for the individual biomarker.

Table S10. Summary of pathways regulated in AECOPD.

Metabolite	Hits[a)]	p value[b)]	Impact factor[c)]
Glycine, serine and threonine metabolism	3	0.0003	0.0726
Synthesis and degradation of ketone bodies	1	0.0224	0
Valine, leucine and isoleucine biosynthesis	1	0.0356	0
Butanoate metabolism	1	0.0659	0
Fructose and mannose metabolism	1	0.0870	0
Pyruvate metabolism	1	0.0954	0
Glycolysis / Gluconeogenesis	1	0.1119	0
Amino sugar and nucleotide sugar metabolism	1	0.1559	0
Arginine and proline metabolism	1	0.1598	0.0121
Aminoacyl-tRNA biosynthesis	1	0.1980	0
Purine metabolism	1	0.2595	0

[a)] Hits mean that the number of matched metabolites in the pathway models; [b)] The p value was calculated by the pathway enrichment analysis of the hypergeometric test; [c)] The pathway impact factor was calculated by pathway topology analysis of relative betweenness centrality.

Reference

(1) Su, H. Y.; Li, X. X.; Huang, L.; Cao, J.; Zhang, M. J.; Vedarethinam, V.; Di, W.; Hu, Z. Q.; Qian, K. Plasmonic alloys reveal a distinct metabolic phenotype of early gastric cancer. *Adv. Mater.* **2021,** *33* (17), 2007978.

(2) Yuan, L.; Hu, Q. Comparisons of three kinds of plane wave methods for the Helmholtz equation and time-harmonic Maxwell equations with complex wave numbers. *J. Comput. Appl. Math.* **2018,** *344*, 323-345.

(3) Werner, W. S. M.; Glantschnig, K.; Ambrosch-Draxl, C. Optical Constants and Inelastic Electron-Scattering Data for 17 Elemental Metals. *J. Phys. Chem. Ref. Data* **2009,** *38* (4), 1013-1092.

(4) Celli, B. R.; Wedzicha, J. A. Update on clinical aspects of chronic obstructive pulmonary disease. *N. Engl. J. Med.* **2019,** *381* (13), 1257-1266.

(5) Huang, L.; Wang, L.; Hu, X.; Chen, S.; Tao, Y.; Su, H.; Yang, J.; Xu, W.; Vedarethinam, V.; Wu, S., et al. Machine learning of serum metabolic patterns encodes early-stage lung adenocarcinoma. *Nat. Commun.* **2020,** *11* (1), 3556-3556.
